# FoxO1 signaling in B cell malignancies and its therapeutic targeting

**DOI:** 10.1002/1873-3468.15057

**Published:** 2024-11-12

**Authors:** Krystof Hlavac, Petra Pavelkova, Laura Ondrisova, Marek Mraz

**Affiliations:** ^1^ Central European Institute of Technology Masaryk University Brno Czech Republic; ^2^ Department of Internal Medicine, Hematology and Oncology University Hospital Brno and Faculty of Medicine Brno Czech Republic

**Keywords:** AS1842856, B cell development, B cell malignancies, chronic lymphocytic leukemia, cpd10, FoxO1, FoxO1 inhibitor, leukemia, lymphoma, targeted therapy

## Abstract

FoxO transcription factors (FoxO1, FoxO3a, FoxO4, FoxO6) are a highly evolutionary conserved subfamily of the ‘forkhead’ box proteins. They have traditionally been considered tumor suppressors, but FoxO1 also exhibits oncogenic properties. The complex nature of FoxO1 is illustrated by its various roles in B cell development and differentiation, immunoglobulin gene rearrangement and cell‐surface B cell receptor (BCR) structure, DNA damage control, cell cycle regulation, and germinal center reaction. FoxO1 is tightly regulated at a transcriptional (STAT3, HEB, EBF, FoxOs) and post‐transcriptional level (Akt, AMPK, CDK2, GSK3, IKKs, JNK, MAPK/Erk, SGK1, miRNA). In B cell malignancies, recurrent *FoxO1* activating mutations (S22/T24) and aberrant nuclear export and activity have been described, underscoring the potential of its therapeutic inhibition. Here, we review FoxO1's roles across B cell and myeloid malignancies, namely acute lymphoblastic leukemia (ALL), acute myeloid leukemia (AML), chronic lymphocytic leukemia (CLL), follicular lymphoma (FL), diffuse large B cell lymphoma (DLBCL), mantle cell lymphoma (MCL), Burkitt lymphoma (BL), Hodgkin lymphoma (HL), and multiple myeloma (MM). We also discuss preclinical evidence for FoxO1 targeting by currently available inhibitors (AS1708727, AS1842856, cpd10).

## Graphical abstract

FoxO1 has context‐specific tumor suppressor or oncogenic character in myeloid and B cell malignancies. This includes tumor‐promoting properties such as stemness maintenance and DNA damage tolerance in acute leukemias, or regulation of cell proliferation and survival, or migration in mature B cell malignancies. FoxO1 activity can be inhibited by small molecules that exhibited promising preclinical activity in hematological malignancies.

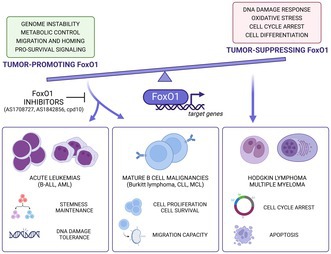

## Abbreviations

**AID**, activation‐induced cytidine deaminase

**Akt**, protein kinase B

**ALL**, acute lymphoblastic leukemia

**AML**, acute myeloid leukemia

**BCR**, B cell receptor

**BL**, Burkitt lymphoma

**BTK**, Bruton tyrosine kinase

**CLL**, chronic lymphocytic leukemia

**CSR**, class‐switch recombination

**DLBCL**, diffuse large B cell lymphoma

**DZ**, dark zone of germinal centers

**FL**, follicular lymphoma

**FoxO**, forkhead box O

**GC**, germinal center

**HL**, Hodgkin lymphoma

**HSC**, hematopoietic stem cell

**IgH/L**, immunoglobulin heavy/light chain

**LZ**, light zone of germinal centers

**MAPK**, mitogen‐activated protein kinase

**MCL**, mantle cell lymphoma

**MM**, multiple myeloma

**PI3K**, phosphoinositide 3‐kinase

**PTEN**, phosphatase and tensin homolog

**SHM**, somatic hypermutation

Fox ‘O’ transcription factors (FoxO1, FoxO3a, FoxO4, FoxO6) represent an evolutionarily conserved subfamily of the ‘forkhead’ box proteins, whose role has been described in processes such as cell cycle regulation, oxidative stress response, stem cell pool maintenance, apoptosis, and others. Traditionally, FoxOs were considered as tumor suppressors since FoxO1, FoxO3a, and FoxO4 triple knockout mice have a strong cancer‐prone phenotype [1]. However, it has recently become evident that FoxOs exhibit remarkable context‐ and tissue‐specific behavior, challenging the general assumption of their tumor‐suppressive role. FoxO1 has been shown to act out of this role more often than other FoxO members, with B cell development being an example of this context‐specific function. The FoxO1 transcriptional programs have various impacts across different stages of B cell development and, in some instances, resemble oncogene‐driven processes (e.g., extensive proliferation of dark‐zone B cells of germinal centers) [2, 3, 4, 5]. FoxO1's bivalent behavior is further reflected in the biology of B cell malignancies. In Hodgkin lymphoma or multiple myeloma, FoxO1 displays a tumor‐suppressive role as it is frequently deleted or mediates therapy‐induced cytotoxicity, respectively [6, 7]. On the other hand, FoxO1's ‘oncogenic’ character has been described in Burkitt lymphoma, mantle cell lymphoma, or chronic lymphocytic leukemia, with malignant B cells utilizing FoxO1 to increase their fitness [8, 9, 10]. In diffuse large B cell lymphoma, *FoxO1* mutations with both activating and inactivating character have been observed [11]. Overall, it has been proposed that FoxO1 inhibition might be a potential therapeutic approach in several hematological malignancies. However, the first FoxO1 inhibition studies originated in diabetes due to its role in glucose and lipid metabolism. In 2010, pilot studies of two small‐molecule inhibitors (*AS1708727*, *AS1842856*) initiated intensive preclinical research of FoxO1 in diabetes [12, 13]. Here, we review the role of FoxO1 in normal and malignant B cells, and the results from preclinical studies of FoxO1 inhibition in hematological malignancies.

## Regulation and function of FoxO transcription factors

**Fig. 1:**
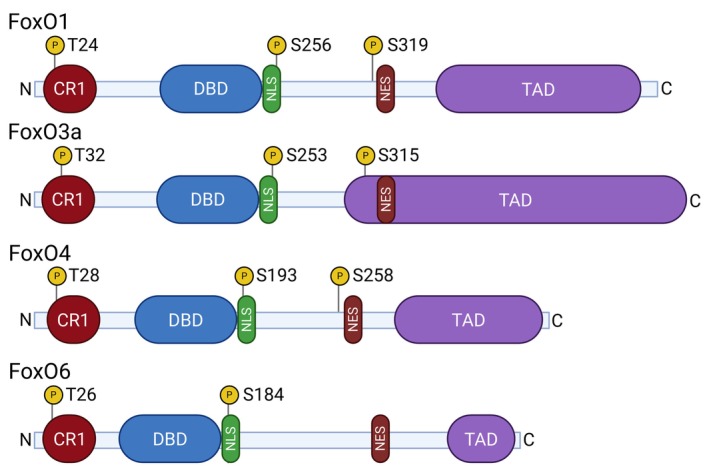
Structure of the FoxO transcription factor family. The phosphorylation sites are highlighted. Phosphorylation of these sites leads to sequestration of FoxO1 by 14‐3‐3 proteins, which impairs binding to DNA and results in the nuclear export of FoxOs. CR1, conserved region 1; DBD, DNA‐binding domain; NES, nuclear export signal; NLS, nuclear localization signal; TAD, transactivation domain.

FoxO transcription factors are one of the 17 subfamilies of the Fox (‘forkhead’ box) protein family, whose common feature is a typical DNA‐binding domain (DBD) structure [14]. This sequence of usually 110 amino acids is composed of three α‐helixes, a short ß‐sheet and two bends that together form the variant helix‐turn‐helix motif, also referred to as a ‘winged helix’ (Fig. 1) [15, 16]. All members of the Fox family bind to DNA as monomers [15]. The ‘O’ subfamily differs from the other subfamilies by a 5 amino acid long insertion between the H2 and H3 helixes. In addition to the 5′‐(A/C)AA(C/T)A‐3′ nucleotide sequence of DNA, which is recognized by all Fox proteins, FoxOs bind to regions consisting of 5′‐GTAAACAA‐3′ sequences (DAF‐16 binding element) and 5′‐(C/A)(A/C)AAA(C/T)AA‐3′ (insulin‐responsive element, IRE). Less evolutionarily conserved regions are NLS and NES (nuclear localization and export signal), which allow regulation of transcription factor transport in and out of the nucleus, and TAD (transactivation domain) with binding sites for regulatory proteins at the C terminus of the protein [17]. The FoxO proteins differ in size, with FoxO1 (*FKHR*) and FoxO3a (*FKHRL1*) lengths exceeding 650 amino acids, while FoxO4 (*AFX*) and FoxO6 are ~ 500 amino acids long due to the reduced size of their TADs (Fig. 1) [18]. The expression levels of the four FoxO family members vary in different cell types, and they have a certain degree of functional redundancy and distinct context‐specific functions [1]. While FoxO1, FoxO3a, and FoxO4 are expressed in virtually all tissues, FoxO6 expression is mainly restricted to the brain [19, 20].

**Fig. 2:**
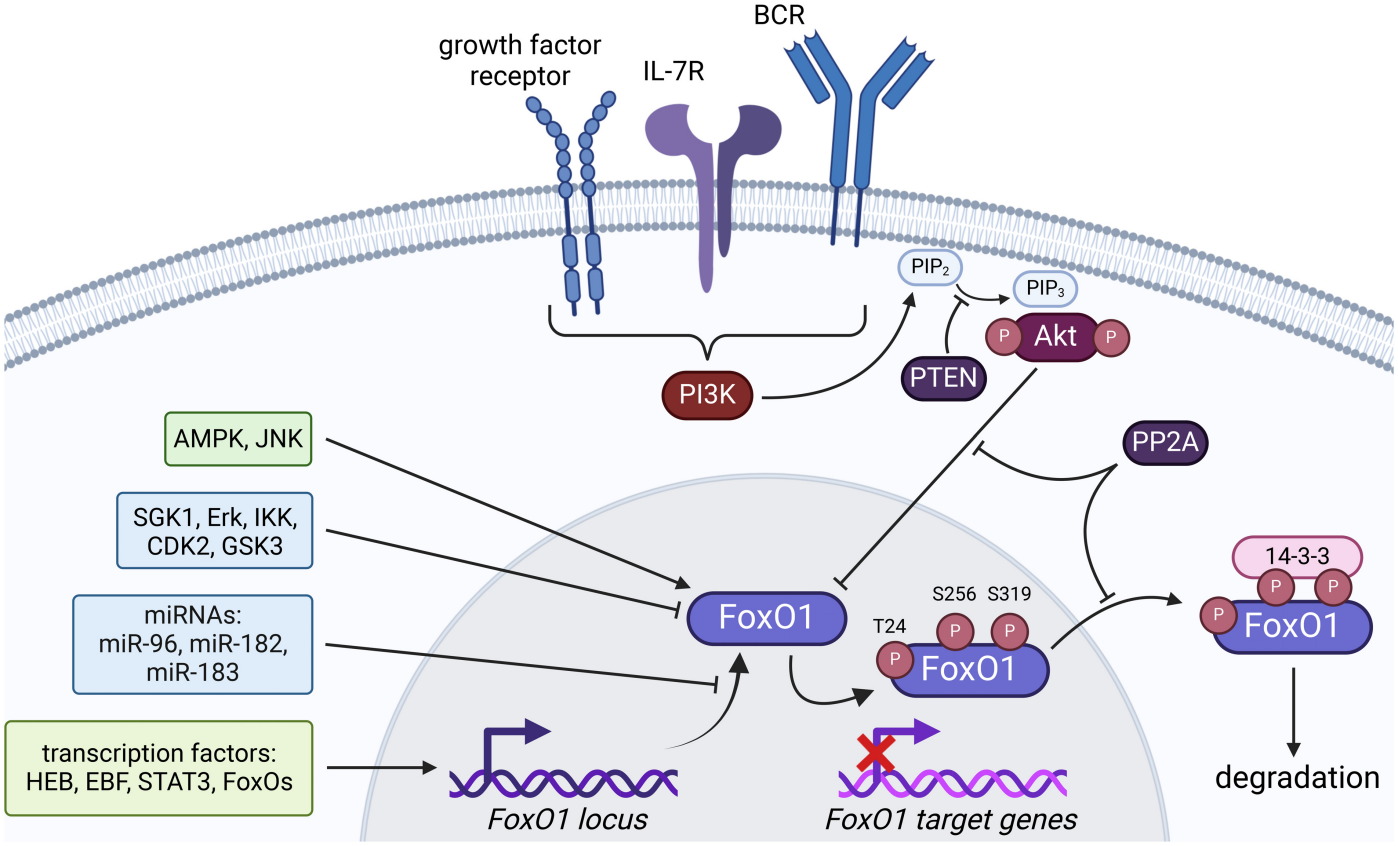
The complex regulation of FoxO1 activity. Transcription of FoxO1 is influenced by transcription factors HEB, EBF, STAT3, and other members of the FoxO family. At the post‐translational level, FoxO1 expression can be inhibited by several miRNAs, namely *miR*‐*96*, *miR*‐*182* and *miR*‐*183*, and *via* phosphorylation by kinases. Kinases can either cause FoxO1 activation (AMPK and JNK), or inhibition (SGK1, MAPK/ERK, IKKs, CDK2, GSK3, and Akt) and subsequent nuclear export and/or degradation. The key player in FoxO1 inhibition is the Akt kinase activated by PI3K upon signals from various cell‐surface receptors.

Strict regulation of the activity of FoxOs is crucial for their correct function and target gene expression (Fig. 2). First, FoxOs are regulated at the transcriptional level by other transcription factors, such as HEB, EBF, and STAT3, but also by FoxOs themselves [21, 22, 23]. MicroRNAs may also limit their expression post‐transcriptionally, for instance, *miR‐96*, *miR‐182*, and *miR‐183* repress *FoxO1* in Hodgkin lymphoma, and *miR‐155* represses *FoxO3a* in Waldenström's macroglobulinemia cells [6, 24, 25]. Phosphorylation plays a central role in regulating the activity of these transcription factors post‐translationally. This can have an activating effect in the case of AMPK or JNK kinases, but also an inhibitory effect caused by phosphorylation mediated by multiple kinases involved in growth factor receptor signaling, namely SGK1, MAPK/Erk, IKKs, CDK2, GSK3, and Akt [26, 27]. Akt, the most studied negative regulator of FoxOs, recognizes and phosphorylates highly evolutionarily conserved regions of FoxOs, containing the RXRXXS/T motif. FoxO1 is phosphorylated by Akt on T24, S256, and S319, FoxO3a on T32, S253, and S315, and FoxO4 on T28, S193, and S258. Akt phosphorylates FoxO6 on T26 and S184 (and lacks the third, C‐terminal phosphorylation site) (Fig. 1). FoxOs are exported from the nucleus upon phosphorylation by Akt, which prevents their transcriptional activity, leading to their cytosolic retention; subsequent degradation in the proteasome is mediated by interaction with 14‐3‐3 proteins [19, 28, 29, 30]. Phosphorylation on the threonine at the N terminus and the first serine near the DBD forms two binding sites bordering the DBD and sterically covering the nuclear localization signal, thereby creating docking sites for 14‐3‐3 proteins. The interaction of 14‐3‐3 with FoxOs abolishes the bond with DNA and blocks reimport into the nucleus; however, the exact structural/conformational changes remain unclear. Exposure of the nuclear export signal and subsequent nuclear export is another mechanism controlling FoxOs' activity through phosphorylation of the mentioned sites (Fig. 2) [26, 31, 32, 33]. In addition, phosphatases are also involved in regulating FoxO activity either directly, such as PP2A phosphatase, or indirectly, such as PTEN phosphatase inhibiting PI3K/Akt signaling (Fig. 2) [34, 35]. The activity of FoxOs is also regulated by other post‐translational modifications, namely ubiquitination, acetylation, and methylation, which can have both activating and inhibitory effects and influence each other [36, 37, 38]. In general, post‐translational modifications play a central role in regulating FoxOs, with phosphorylation by Akt being the most canonical.

FoxOs are often considered negative cell cycle regulators, and their activation can occur in response to stress stimuli such as DNA damage or oxidative stress (mediated by AMPK or JNK kinases). Cells can utilize FoxOs to initiate a stress response that ensures survival and enhances their overall fitness. FoxO‐mediated cell cycle arrest is driven by the repression of cyclin D1/2 [39, 40] and the induction of cyclin G2, p21^Cip1^, p27^Kip1^, and Rb2/p130 [41, 42]. FoxOs' protective role is then displayed *via* FoxO‐dependent ATM, GADD45α, or SOD2 and catalase induction to address either DNA damage or ROS presence in the cell [43, 44]. A decisive tumor‐suppressive role of FoxOs is present upon cellular damage, which results in FoxO‐mediated induction of pro‐apoptotic genes such as *TNFSF10* (TRAIL), *FASLG* (FasL), *BCL2L11* (BIM), or *BBC3* (PUMA) [29, 45, 46, 47]. In line with this, FoxO inhibition is required for cell cycle progression, which is mediated by various effectors. In the case of G1/S transition, these include Akt, MAPK, CDK2, and other kinases activated in response to growth factors [6, 48, 49]. During the G2/M checkpoint, FoxOs' major regulators are CDK1 and Skp2, which mediate phosphorylation and subsequent ubiquitination, respectively, following successful DNA integrity control [50, 51, 52].

Cell cycle arrest plays a crucial role in maintaining the stem cell pool by allowing cells to remain quiescent (reviewed in [53]). The contribution of FoxOs to stemness maintenance is mainly present in adult stem cells, which serve as a source for the regeneration and renewal of differentiated tissues. FoxOs promote quiescence of neural, myogenic, and hematopoietic stem cells, which is primarily mediated by the expression of cell cycle regulators mentioned above (in the case of neural and hematopoietic cells [43, 54]), but also by repression of differentiation factors (Notch signaling modulation in myogenic cells and *ASCL1* repression in neural cells [55, 56]), or by metabolic stress control and ROS monitoring [57, 58]. Apart from adult stem cells, FoxOs (especially FoxO1) regulate the pluripotency of embryonic stem cells *via* direct *OCT4* and *SOX2* transcriptional induction [59]. Additionally, embryonic stemness and pluripotency depend on high autophagic flux and proper proteasome function, where FoxO1 and FoxO4 display major regulatory roles [60, 61].

FoxOs can also impact cell differentiation in a context‐ and cell‐type‐dependent fashion. FoxO‐specific transcriptional programs change during various stages of differentiation, and FoxO members can alternate during the cell differentiation process. In hematopoiesis, FoxO1 drives mostly lymphoid cell differentiation, whereas FoxO3a is required for proper myeloid development [2, 62]. A similar situation occurs during osteogenic differentiation, where, at first, FoxO1/3a promote transition from mesenchymal stem cells to early osteogenic progenitors. Later, however, FoxOs prevent these progenitors from differentiating into osteoblast precursors [63, 64]. FoxOs were also shown to mediate terminal stages of chondrogenesis and adipogenesis [65, 66, 67]. This review further describes FoxO1's role in B cell development and discusses how these functions can influence malignant transformation.

## FoxO1 in the development and functions of normal B cells

**Fig. 3:**
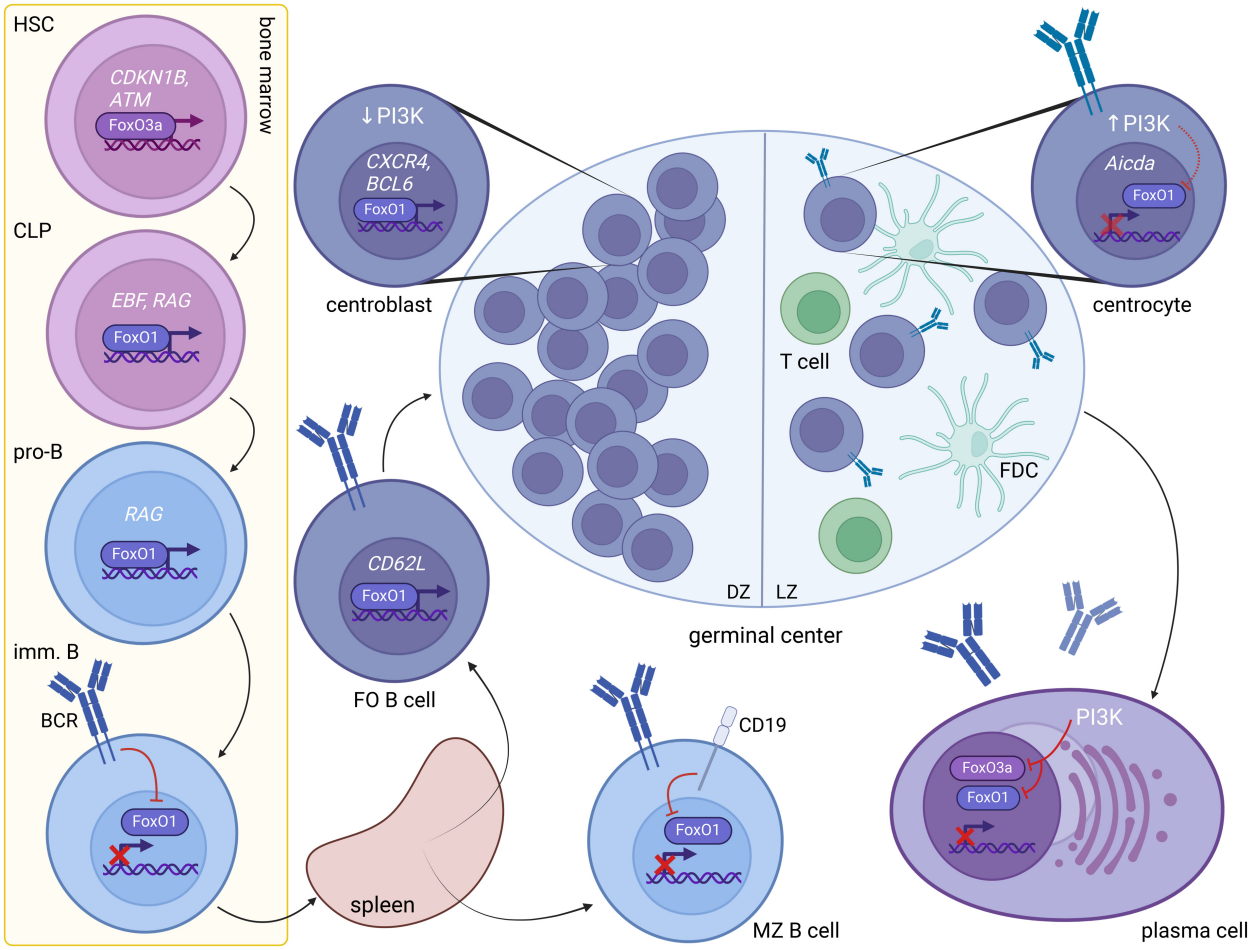
FoxO1 involvement in B cell development with examples of its transcriptional targets typical for specific cell developmental stages. FoxO3a is crucial at the HSC stage in the maintenance of a stable HSC pool. FoxO1 takes over FoxO3a at the CLP stage, assuring B cell lineage commitment. Its activity is indispensable for proper V(D)J rearrangement to establish unique B cell receptors (BCRs). Furthermore, FoxO1 contributes not only to follicular B cell homing into germinal centers (GC) but also to the formation and polarization of these microanatomical structures. FoxO1 supports the clonal proliferation of activated B cells after antigen recognition and their positive selection and affinity maturation of antibodies. Differentiated plasma cells show less dependency on FoxO1 activity, where simultaneous inhibition of FoxO1 and FoxO3a *via* PI3K activity is necessary for plasma cell survival. CLP, common lymphoid progenitor; DZ, dark zone; FDC, follicular dendritic cell; FO B cell, follicular B cell; HSC, hematopoietic stem cells; imm. B, immature B cell; LZ, light zone; MZ B cell, marginal zone B cell.

The development of B lymphocytes (summarized in Fig. 3) is an intricate process that requires strict and precise regulation of specific genes, in which FoxOs, particularly FoxO1 and FoxO3a, are involved in cooperation with other transcription factors. In hematopoietic stem cells (HSC), FoxO3a has a ‘superior’ role compared to FoxO1, and its continuous activity maintains an HSC pool *via* cell cycle arrest (p27^Kip1^) and controls HSC survival *via* oxidative stress and DNA damage monitoring (ATM) [68, 69]. At the common lymphoid progenitor (CLP) stage, a transition from predominant FoxO3a expression to FoxO1 occurs, which, in cooperation with E proteins (E2A, HEB), EBF and PAX5, directs the further development of CLP towards the B cell lineage and differentiation to the pro‐B lymphocyte stage [2, 22]. The importance of FoxO1 in CLP lineage commitment is further supported by experiments with HSC engineered for combined *FoxO1* and *FoxO3a* loss, in which HSC failed to differentiate further into the lymphoid cells (Fig. 3) [70].

At the pre‐B cell stage, FoxO1 induces expression of recombinases *RAG1* and *RAG2*, both by direct binding to the *RAG* locus and its enhancer region and by inducing expression of other target genes (e.g., the gene for GADD45α), which subsequently influence *RAG* expression. RAG1 and RAG2 are responsible for rearranging V(D)J segments of immunoglobulin heavy chain (IgH) [71, 72]. After successful recombination, RAG activity is temporarily suppressed by IL‐7R signaling, which activates the PI3K/Akt axis and inhibits FoxO1. The second wave of RAG1/2 activity occurs in pre‐B lymphocyte during VJ recombination of immunoglobulin light chain (IgL). Similarly, after successfully assembling cell‐surface immunoglobulin, that is, B cell receptor (BCR), FoxO1 is again inhibited by the newly emerged BCR signaling cascade, halting the IgH/L recombination process [2]. However, *RAG* expression does not stop when immature B cells express autoreactive BCR. In this case, the receptor is internalized, inhibiting PI3K/Akt signaling, which allows for FoxO1 activation and, therefore, *RAG* expression for additional rounds of recombination, that is, receptor editing (Fig. 3) [71].

Mature B lymphocytes, expressing a unique BCR on their surface, represent the late stage of antigen‐independent B cell development. Continuous ‘tonic’ BCR signaling is required for their survival outside the bone marrow, and surface BCR deletion leads to apoptosis [3, 73]. However, genetic BCR ablation can be compensated for by constitutive downstream signaling, particularly by PI3K activity. Artificially induced PI3K/Akt signaling ensures the survival of BCR‐deleted B cells *via* reducing the amount of FoxO1 in the nucleus, which therefore does not induce antiproliferative genes (e.g., cyclin G2 or Rb2/p130), and a similar phenotype rescuing BCR‐negative B cells was observed by *FoxO1* knockout [3, 74, 75]. In mature B lymphocytes, FoxO1 also contributes to directing their further development into distinct subpopulations, since its ectopic activity (caused by either Akt or CD19 knockout or deletion of the catalytic subunit of PI3K) leads to reduced numbers of marginal zone (MZ) and B1 cells (Fig. 3) [76, 77]. In line with these observations, *FoxO1* or *PTEN* deletions, as well as constitutively active Akt promote MZ cell differentiation at the expense of follicular B cells, underscoring that Akt activity and FoxO1 localization are key factors determining the formation of these subpopulations [77, 78, 79]. In follicular B cells, the activity of PTEN and its effector FoxO1 is also relevant, as it is responsible for increased IgD expression compared to IgM. However, this is a result of FoxO1 interactions with other transcription factors that affect IgD pre‐mRNA splicing [80]. Last but not least, FoxO1 positively influences mature B cell migration into lymph nodes *via* L‐selectin (CD62L) induction (Fig. 3). *FoxO1* deletion in B cell developmental stages beyond pro‐B stage results in decreased B cell numbers in lymph nodes by approximately 30% [2].

During developmental processes induced in response to antigen recognition by BCR, FoxO1 plays an irreplaceable role. Upon initial antigen recognition, naïve B cells get activated by T helper cells, proliferate, and form germinal centers (GC). Massively proliferating B cells (centroblasts) represent the dark zone (DZ), where they expand and attempt to increase the diversity and affinity of their BCRs *via* somatic hypermutations (SHM). Then, B cells with ‘rebuilt’ BCRs migrate as centrocytes to the light zone (LZ), where the affinity is ‘tested’. Centrocytes with a suitable BCR affinity for antigen are positively selected for further differentiation *via* T helper cell interactions. These interactions in the LZ also determine whether B cells undergo class‐switch recombination (CSR). Alternatively, B cells with insufficient antigen affinity might re‐enter the DZ for additional rounds of SHM to refine their BCR. Generally, FoxO1 is involved in GC formation, maintenance, and its polarization. Reflecting the different roles of DZ and LZ in the process of affinity maturation, FoxO1 activity varies between these two compartments, as it has been observed to be transcriptionally active in DZ cells and inhibited by increased PI3K signaling in LZ cells [4, 5]. Contrary to FoxO1's antiproliferative behavior in other contexts, in DZs, FoxO1 induces *BCL6*, which is responsible for enhanced B cell proliferation (Fig. 3) [5]. In line with this, *FoxO1* knockout in GC B cells in mouse models leads to a lack of B cells with a typical ‘DZ‐phenotype’ (CXCR4^high^, CD86^low^, CD83^low^), and in addition, GCs consist of only ‘LZ‐like’ cells [81]. CXCR4 is a key FoxO1 transcription target, and its absence disrupts B cells' ability to enter the DZ from the LZ. Moreover, GC B cells with *FoxO1* deletion have a slower cell cycle, and their proliferation could not be further induced by supporting signals from T lymphocytes [4, 81, 82].

Centroblasts that have undergone clonal expansion in the DZ further exhibit the mutagenic activity of AID, whose expression is controlled by FoxO1. AID converts cytosine to uracil, leading to SHM, which increases the antibody diversity and subsequently contributes to higher antibody affinity. Interestingly, SHM is not fully dependent on FoxO1 activity despite being regulated by the activity of FoxO1's target, AID. *FoxO1* knockout cells exhibit fewer Ig mutations than in control cells, suggesting that other factors (such as HoxC4) coregulate SHM [2, 4, 5, 83]. On the other hand, during CSR in the LZ, the purpose of which is to alter antibodies' effector function, FoxO1 engagement in regulating AID expression is of far greater importance (Fig. 3). Consistent with the mechanism of FoxO1‐negative regulation by Akt, it was observed that inhibiting PI3K increases CSR frequency, while CSR was halted in cells expressing constitutively active Akt or harboring *PTEN* or *FoxO1* deletion [2, 84].

Centrocytes, B cells with sufficient affinity for the antigen achieved through SHM, are positively selected in the LZ to further develop into plasma and memory B cells. This selection also seems to be influenced by FoxO1 activity. Activating *FoxO1* mutations (see below) change transcription targets and also cause increased sensitivity to stimuli from helper T lymphocytes. This has implications for the biology of GC‐derived B cell lymphomas, where both FoxO1 signaling and positive selection are often deregulated [85]. After differentiation into plasma or memory B cells, the role of FoxO1 does not seem to be essential, while the involvement of FoxO3a is more prominent. Active PI3K signaling inhibits both FoxO1 and FoxO3a and ensures plasma cell survival [86].

In summary, FoxO1 has a key role in the development of B cells, with its function varying significantly between immature and mature B cells (Fig. 3). In immature B cells, FoxO1 is essential for driving processes like V(D)J recombination by inducing the expression of *RAG* genes critical for the immunoglobulin rearrangement. During this stage, FoxO1 activity is tightly regulated by PI3K/Akt signaling, which temporarily inhibits FoxO1 after successful BCR assembly. In mature B cells, FoxO1's role shifts towards influencing cell survival, differentiation into subpopulations, and migration. Furthermore, FoxO1 regulates the proportional balance of follicular and marginal zone B cells. Upon activation by antigen recognition, FoxO1 supports B cell proliferation in the dark zone of germinal centers, a role that contrasts with its typical antiproliferative functions in immature B cells. Also, FoxO1 contributes to proper antibody affinity maturation during both SHM and CSR. The context‐dependent activity of FoxO1 is likely linked to its various ‘oncogenic’ and ‘tumor‐suppressive’ functions in B cell neoplasms.

## FoxO1 in malignant B cells

**Fig. 4:**
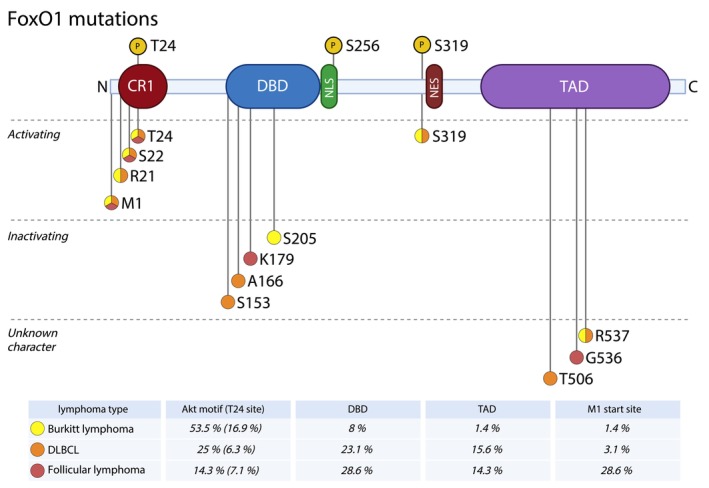
*FoxO1* mutations in B cell malignancies. In classical Hodgkin lymphoma, complete *FoxO1* loss occurs due to deletion of the 13q14 locus in ~ 11% of cases. In Burkitt lymphoma (BL), follicular lymphoma (FL), and diffuse large B cell lymphoma (DLBCL), *FoxO1* is frequently affected by point mutations (up to 54% cases in BL, 5% in FL, and 8.6% in DLBCL). The figure depicts the most frequent mutation hotspots in the *FoxO1* gene. The table below displays the percentage distribution of *FoxO1* mutations across different protein domains/functionally important sites, indicating the relative proportion of mutations found within each specific region [8, 11, 156, 157].

In B cell malignancies, it has been suggested that the FoxO1 function will correspond to its role in the healthy developmental counterpart of the B cell malignancy. This assumption is likely correct in several cases (acute pre‐B cell leukemia, Burkitt lymphoma, or multiple myeloma), but is not universal. In other B cell malignancies, such as chronic lymphocytic leukemia, diffuse large B cell lymphoma or mantle cell lymphoma, FoxO1 can exhibit functions that diverge from those seen in their healthy counterparts. Given the intriguingly complex role of the FoxO1 transcription factor in B cell physiology, it is not surprising that malignant B cells attempt to modulate or utilize its functions to increase their fitness by both genetic alterations and aberrant FoxO1 regulation (recurrent *FoxO1* mutations are summarized in Fig. 4). In some diseases, such as Hodgkin lymphoma or multiple myeloma, FoxO1 plays its role as a tumor suppressor and neoplasia development is characterized by a lack of FoxO1 activity. However, in diffuse large B cell lymphoma, malignant cells utilize FoxO1 in contradictory ways with recurrent presence of both activating and inactivating mutations. In Burkitt lymphoma, mantle cell lymphoma, follicular lymphoma, and chronic lymphocytic leukemia or acute lymphoblastic leukemia, FoxO1 exhibits a key oncogenic character that allows cell survival, suggesting the possibility of its therapeutic targeting.

Classical Hodgkin lymphoma (cHL) resembles differentiated, post‐GC B cells with its transcription program. This similarity is also evident in terms of FoxO1 expression or activity, which are regulated at several levels in cHL: by PI3K/Akt and MAPK/Erk signaling cascades, or by *miR‐96*, *miR‐182* and *miR‐183* [6]. FoxO1 inhibition not only eliminates its pro‐apoptotic activity, but the absence of its activity is necessary to prevent PRDM1 expression which would lead to the induction of plasma cell differentiation. In addition, ~ 11% of cHL patients harbor large 13q14 deletions, including the *FoxO1* locus [6]. Physiologically, however, FoxO1 does not appear to be involved in plasma cell differentiation, unlike FoxO3a [87]. Similarly, primary mediastinal B lymphoma, a rare non‐Hodgkin lymphoma subtype histologically partially similar to cHL, is also characterized by a decrease in FoxO1 levels. Here, FoxO1 is negatively regulated by hypermethylation of its promoter and *via* Myc activity [88].

Another example of FoxO1's tumor‐suppressive role is observed in multiple myeloma (MM), a plasma cell malignancy. Several studies have demonstrated that the cytotoxic effects of Akt or GSK3 inhibitors are specifically mediated by the activation of both FoxO1 and FoxO3a [7, 89]. Additionally, selinexor, a recently approved nuclear export inhibitor for MM therapy, is thought to retain FoxO1 and FoxO3a in the nucleus as one of its major mechanisms of action [90, 91]. Furthermore, MM patients with high FoxO‐suppressed gene expression (reflecting a lack of FoxO1 activity) exhibit inferior overall survival [7].

Diffuse large B cell lymphoma (DLBCL), a heterogenous lymphoma entity, can exhibit complex FoxO1 (in)activity. FoxO1 inhibition *via* PI3K/Akt signaling has been described, which can be further enhanced by the relatively common loss of *PTEN* [92, 93]. Moreover, *miR‐21*, which is abundantly expressed in DLBCL, also directly targets FoxO1, contributing to its downregulation. In this case, *miR‐21*‐mediated repression of FoxO1 leads to down‐modulation of the levels of BIM, a pro‐apoptotic member of the BCL2 family [94]. FoxO1 tumor suppressor activity in some DLBCL cases is further supported by the fact that its ectopic activity, induced by Akt or SYK inhibitors, leads to apoptosis induction in these cells. This was linked to an indirect, FoxO1‐mediated induction of another pro‐apoptotic BCL2 family protein, HRK [95]. In this context, however, FoxO1, which is activated upon SYK inhibition, transcriptionally induces *SYK* and *CD79B* and therefore is potentially involved in the rewiring of the BCR signalosome and compensatory response to the drug [96]. Additionally, *FoxO1* mutations are found in approximately 8.6% of DLBCL cases (Fig. 4) [11]. In line with the aforementioned studies, ~ 39% of these mutations are loss‐of‐function mutations in the DBD domain. However, DBD‐mutated FoxO1 preserves its cytoplasmic function and has been hypothesized to promote functions beneficial for lymphoma B cells [97]. Notably, directly opposing the concept of FoxO1 being a tumor suppressor, ~ 46% of *FoxO1* mutations involve sites close to T24, a phosphorylation site for negative regulation, making this site unrecognizable for Akt, and subsequently reducing FoxO1 nuclear export. Recently, it has been confirmed that T24‐related mutations drive the FoxO1 oncogenic transcriptional program, which differs from wild‐type FoxO1 [98]. The oncogenic FoxO1 activity in DLBCL is not dependent on mutations in all cases since FoxO1's transcriptional activity following PTEN activation induces the expression of the anti‐apoptotic protein BCL2 [99].

In follicular lymphoma (FL), usually an indolent disease of GC centrocytes, *FoxO1* mutations occur with an incidence of about 5% and up to 15% in FL transformations to DLBCL (Fig. 4) [100]. However, the functional consequences of these mutations in FL have not yet been thoroughly investigated. They are considered as gain‐of‐function leading to BCL6 upregulation and subsequent BCL6‐mediated suppression of p53 [101]. Disrupting p53 function has been previously described as one of the key mechanisms in FL histological transformation [100, 102]. Since these mutations are associated with a poorer prognosis, the *FoxO1* mutation status is included in the m7‐FLIPI prognostic scoring system [100, 103, 104].

**Fig. 5:**
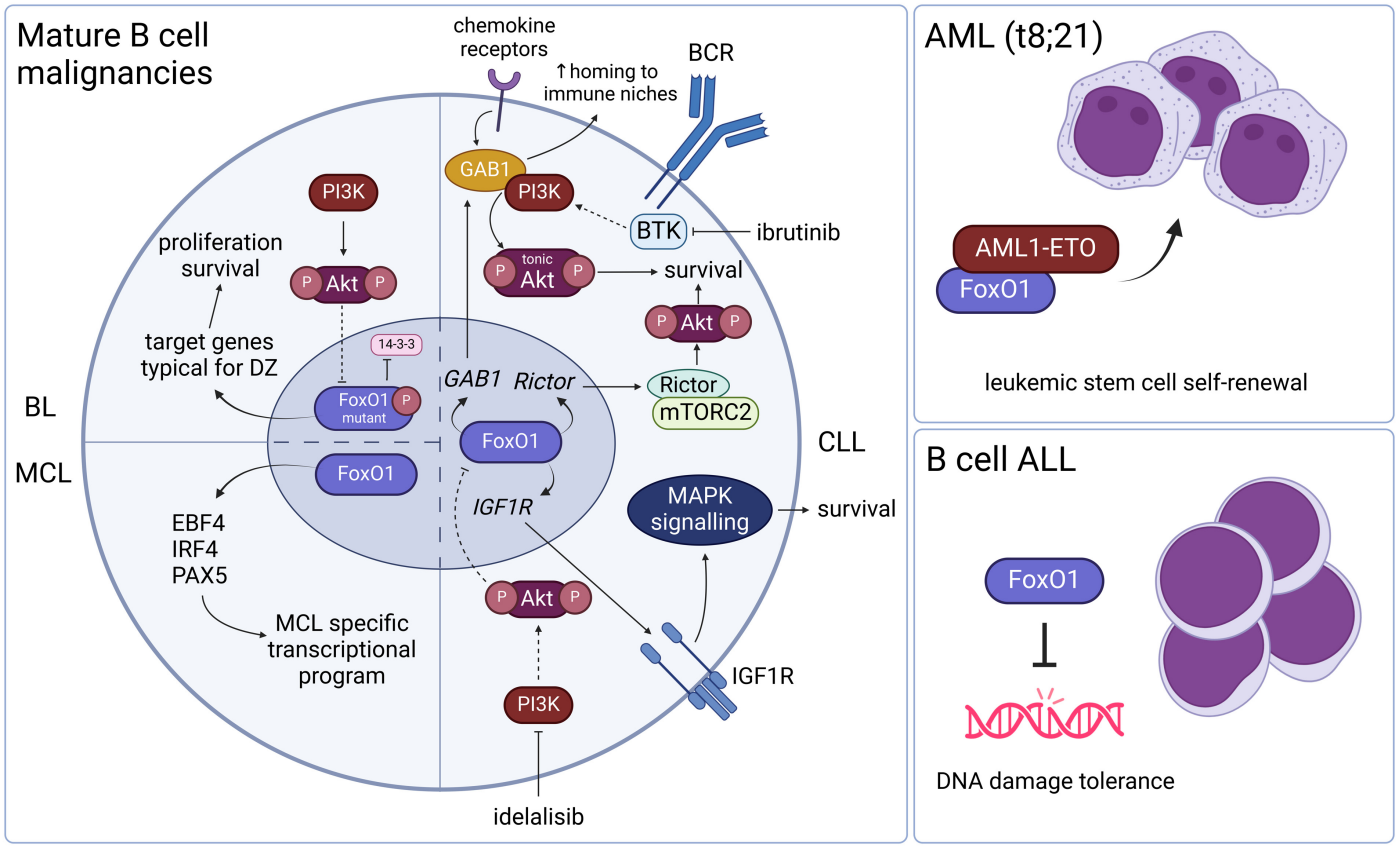
Oncogenic role of FoxO1 in hematological malignancies. In Burkitt lymphoma (BL), mutated FoxO1 is unresponsive to inhibiting Akt phosphorylation; therefore, PI3K/Akt signaling and transcriptionally active FoxO1 co‐occur. Here, FoxO1 induces the expression of genes characteristic for the dark zone (DZ) of germinal centers, promoting BL cell proliferation. In mantle cell lymphoma (MCL), the activity of several transcription factors is essential, with FoxO1 representing a master regulator of their expression. In chronic lymphocytic leukemia (CLL), FoxO1 contributes to the survival of circulating CLL cells and increases the homing capacity of circulating CLL cells back into the CLL microenvironment. FoxO1 also contributes to the adaptation to therapy by BCR signaling inhibitors. Upon PI3K inhibition by idelalisib, FoxO1 induces IGF1R expression, subsequently activating pro‐survival MAPK signaling. In the case of BTK inhibitors, FoxO1 upregulates GAB1, which triggers ‘tonic’ Akt signaling. Moreover, FoxO1 also induces Rictor, a member of the mTORC2 complex, and thus promotes pro‐survival Akt activity and adaptation to BTK inhibition by two independent mechanisms. In acute hematological malignancies, FoxO1 contributes to preleukemic stem cell self‐renewal, as described in one of the AML subtypes, and also to susceptibility of malignant lymphoblasts to genetic lesions observed in B cell ALL.

Burkitt lymphoma (BL) shares significant biological similarities with the centroblast stage of B lymphocyte development, as BL histologically closely resembles the dark zone of GC and the clonal expansion of B cells. A hallmark of BL is the high expression and sustained activity of FoxO1, which reflects the physiological DZ program. Despite active PI3K/Akt signaling, FoxO1 remains transcriptionally active. This is largely due to mutations at key Akt recognition motifs in FoxO1 (e.g., S22, T24; Fig. 4), observed in 39% of sporadic BL cases and 54% of endemic BL cases [8]. While Akt signaling alone drives cell proliferation, FoxO1 further amplifies clonal expansion in a manner similar to its normal germinal center activity (Fig. 5). Interestingly, it seems that this is not limited to cases with *FoxO1* mutations, and it is specifically the maintenance of FoxO1's activity in the nucleus that increases BL cell fitness. However, the precise molecular mechanism behind the retention of non‐mutated FoxO1 in the cell nucleus remains unclear [8, 105]. Paradoxically, unveiling another layer of complexity, artificial FoxO1 hyperactivation in BL cells has been shown to cause cell cycle arrest and increase apoptosis, highlighting the importance of ‘just right’ levels of FoxO1 activity for malignant cells to maintain their survival [106].

In mantle cell lymphoma (MCL), an aggressive lymphoma of pre‐GC cells, FoxO1 has been recently shown to play a master regulatory role, which exhibits certain properties reminiscent of the FoxO1‐driven B cell lineage commitment program. EBF1, PAX5, and IRF4 transcription factors guided by FoxO1 have been described to induce MCL lineage‐survival transcriptional programs, similarly to how these factors cooperate during the early stages of B cell development (Fig. 5) [9]. FoxO1 interaction with p300 histone acetyltransferase allows for chromatin relaxation and subsequent induction of the MCL‐specific transcriptional program. Additionally, the co‐occurrence of high levels of FoxO1 and active PI3K/Akt is usually found in MCL, suggesting uncoupled FoxO1 regulation (without the presence of *FoxO1* mutations) [9]. This pattern of impaired FoxO1 control occurs in multiple B cell malignancies, but the molecular mechanism remains unclear.

Chronic lymphocytic leukemia (CLL) represents another malignancy of mature B cells where FoxO1 has tumor‐supporting functions (Fig. 5). In CLL, no *FoxO1* mutations have been discovered, but FoxO1 is upregulated in CLL cells compared to healthy B cells [107]. FoxO1 has been shown to drive the expression of GAB1 (an adaptor protein interacting with PIP3 and PI3K) in CLL cells isolated from peripheral blood. This helps CLL cells to maintain ‘tonic’ PI3K/Akt pro‐survival signaling and enhances their chemokine‐guided homing capacity towards the lymph nodes, where they obtain signals required for proliferation (Fig. 5) [108, 109, 110, 111]. FoxO1's oncogenic character also emerges during BCR inhibitor therapy with BTK or PI3K inhibitors. We and others have recently shown that FoxO1 is induced during BCR inhibitor therapy and supports CLL cells' survival [10, 108, 112]. FoxO1 restores Akt activity during BTK inhibitor treatment by two distinct mechanisms. The first is GAB1 induction, which supports the activity of PI3K that bypasses BTK inhibition [10, 108]. Secondly, FoxO1 upregulates Rictor, a key assembly protein of the mTORC2 complex. FoxO1‐mediated upregulation of Rictor subsequently leads to direct Akt phosphorylation (at serine 473), providing a pro‐survival adaptation to BTK inhibition (Fig. 5) [10]. Notably, compensatory Akt activation during BTK inhibitor treatment did not interfere with nuclear FoxO1 localization or trigger its degradation [10]. However, other groups reported that FoxO1 exhibited high levels of inhibitory phosphorylation in some CLL samples, suggesting that in some situations, it might be mostly inactive [107]. FoxO1 is inactivated in CLL cells in the context of continuous and robust Akt activation by microenvironmental stimuli that tips the balance towards its degradation [10, 108]. Overall, CLL cells can have both nuclear FoxO1 activity and ‘tonic’ Akt activity, but strong microenvironmental stimuli driving extensive Akt activation will eventually lead to FoxO1 degradation [10].

The last evidence of FoxO1's oncogenic potential comes from the field of B cell precursor acute lymphoblastic leukemia (BCP‐ALL), the most common malignancy in children. In BCP‐ALL, the constitutive activity of PI3K/Akt, MAPK/Erk, and NF‐κB pathways is well‐described. These pathways inactivate FoxO1, mediate its nuclear export, and promote the survival of transformed pre‐B cells [113, 114]. Paradoxically, it has also been shown that malignant pre‐B cells maintain a substantial portion of nuclear FoxO1. This active FoxO1 drives transcription of *RAG1/2* and *Aicda (AID)* genes, allowing low‐level susceptibility to genetic lesions, a factor driving clonal ALL cell evolution (Fig. 5) [113, 115]. The relevance of FoxO1 in ALL cells was underscored by a study demonstrating that cell growth and viability were reduced by FoxO1 inactivation *via* modulating its upstream regulators, namely *PTEN* (a PI3K activity antagonist) knockout or introduction of constitutively active SYK or Akt. This phenotype resembled a response to autoreactive pre‐BCR, suggesting FoxO1 preserves its physiological function, which is precisely fine‐tuned for the needs of malignant lymphoblasts [116].

In summary, the role of FoxO1 in B cell malignancies, such as BL, MCL, CLL, and BCP‐ALL, reveals a complex involvement in promoting cell survival, proliferation, and disease progression. However, dysregulation of FoxO1 can also induce cell cycle arrest and apoptosis under certain conditions, highlighting the delicate balance of FoxO1 activity required for malignant cells to thrive. Whether it is *via* promoting tonic signaling, regulating transcription factors essential for cell survival, or maintaining stem‐like properties, FoxO1 plays a role in driving the malignant phenotype across various B cell malignancies. Recent efforts have focused on developing strategies to inhibit FoxO1 activity in selected B cell malignancies.

## FoxO1 as a druggable protein

Apart from B cell development and related malignancies, FoxO1 has been primarily studied for its importance in glucose and lipid metabolism. The idea of targeting FoxO1 originated from its studies in diabetes two decades ago. Targeting FoxO1 has clinical relevance, especially for patients with type II diabetes, whose cells cannot metabolize glucose from the blood stream due to either ineffective signaling downstream of the insulin receptor or insufficient insulin production in pancreatic β cells. In healthy cells, FoxO1 is deactivated by Akt phosphorylation as a response to insulin presence in blood. This FoxO1 repression causes a shift in the metabolic program towards utilizing glucose from the blood rather than generating it from noncarbohydrate sources, which happens *via* downregulation of gluconeogenic enzymes like G6Pase, fructose‐1,6‐biphosphatase, and PEPCK at the transcriptional level [37, 117, 118]. The first *in vivo* study suggesting the relevance of FoxO1 inhibition in diabetes was published in 2003 [119]. The authors identified FoxO1 as a key factor driving glucose production in hepatic tissue, ultimately leading to fasting hyperglycemia in diabetic mice. They demonstrated a significant reduction in blood glucose levels by introducing an inactive FoxO1 variant (FoxO1‐∆256) into the hepatic tissue of db/db mice (a common murine model for studying diabetes, obesity, and metabolic disorders, due to a mutation in the leptin receptor gene). Two simultaneous publications in 2010, led by the same research group, described two novel small‐molecule inhibitors of FoxO1 DNA‐binding activity and tested their effects in the db/db mouse model. Nagashima *et al*. [12] described the discovery of *AS1842856*, whereas Tanaka *et al*. [13] synthesized *AS1708727*. These studies, similar in design, showed the inhibitors' potent effect on reducing glucose production in the liver of diabetic mice, while neither insulin nor glucose levels were affected in normal mice. The effect of these inhibitors was assessed based on FoxO1 transactivation using luciferase reporter assay with a ‘four‐repeated’ IRE construct. Furthermore, mRNA levels of *G6Pase* and *PEPCK* served as markers of the effectivity of the inhibitors. Overall, *AS1842856* had lower IC_50_ for FoxO1 inhibition (0.033 μm according to the luciferase assay vs. an IC_50_ of 0.33 μm for *AS1708727*). However, 0.1 μm AS1842856 concentration also inhibited FoxO3a and FoxO4 (3% and 20% inhibition of transactivation activity, respectively). Inhibition of FoxO1 with *AS1842856* or *AS1708727* compounds was not affected by protein phosphorylation or acetylation, and FoxO1 inhibition did not impact nucleus/cytoplasm transport [12, 13].

Targeting FoxO1‐dependent regulation of gluconeogenesis in the liver appeared to be a highly feasible approach for diabetes treatment. However, besides reducing glucose utilization in the tissue, FoxO1 also represses *de novo* lipogenesis; thus, when FoxO1 is inactivated, lipogenesis is induced. Additionally, FoxO1 genetic inactivation exacerbated lipid metabolism abnormalities during hyperglycemia [120, 121, 122]. These observations are crucial for insulin‐treated patients, as weight gain is a common side effect. Therefore, the importance of FoxO1 in lipid metabolism has shifted development towards more specific modulators of FoxO1 activity, aiming to inhibit gluconeogenesis while preserving FoxO1's role in repressing lipid metabolism. A high‐throughput screening study revealed several small molecules that bind FoxO1 and might be suitable for such a strategy [123]. In recent years, while research in the field of diabetes has focused on alternative ways to utilize the vast knowledge on this transcription factor, FoxO1 became a relevant target in several hematological malignancies as well as in some solid tumors (see below).

## Targeting FoxO1 in hematological malignancies

FoxO1 inhibition in cancer requires a robust assessment of the inhibitors' specificity and potential toxicity. So far, preclinical testing has been performed in acute leukemias and mature B cell malignancies and recently in some solid tumors. The majority of experiments were performed using the strategy of FoxO1 inhibition *via* the disruption of FoxO1's DNA‐binding ability, but novel approaches were also described, such as inhibiting FoxO1 co‐activating interaction or blocking FoxO‐specific DNA elements.

The first steps towards testing FoxO1 inhibition in cancer were conducted in acute myeloid leukemia (AML). Here, maintaining stemness is the key process in which FoxO proteins were studied. FoxO1 and its inhibition were investigated in an AML subtype characterized by translocation t(8;21), leading to the expression of fused oncogene AML1‐ETO. In this context, FoxO1 drives preleukemic stem population self‐renewal in cooperation with AML1‐ETO (Fig. 5), while administering *AS1842856* (0.1 μm) almost completely represses preleukemic cell growth with a minimal effect on healthy human CD34+ hematopoietic stem cells *in vitro*. Additionally, FoxO1 inhibition reduced the colony formation ability of patients' myeloid blasts [124]. In acute promyelocytic leukemia, FoxO3a has been shown to be a key effector in differentiation‐based therapy by all‐*trans* retinoic acid, whereas in mixed‐lineage AML, FoxO3a activity favors disease progression, and its high expression is associated with AML stem cell pool expansion [125, 126, 127]. Targeting FoxOs remains an active topic in AML, including new approaches for disrupting FoxO transcriptional networks, such as blocking FoxO‐specific DNA elements [128]. This study developed gene‐specific DNA‐binding pyrrole‐imidazole polyamides (PIPs) to block FoxO‐responsive elements to inhibit FoxO3a‐driven *TRIB1* expression, a key factor in maintaining AML cells' stemness. PIPs were additionally conjugated with chlorambucil and effectively induced AML cell differentiation and cell death [128].

The first B cell‐related study of FoxO1 inhibition was performed in B cell precursor acute lymphoblastic leukemia (BCP‐ALL), where the fine‐tuned FoxO1 activity allows for low‐level DNA damage susceptibility and clonal ALL cell evolution (Fig. 5) [113, 114, 115, 116]. These observations resulted in preclinical testing of FoxO1 inhibition by *AS1842856*. Administering FoxO1 inhibitor (dosed at 20 to 320 nm *in vitro* and 50 mg·kg^−1^·day^−1^ *in vivo*) showed significant cytotoxicity *in vitro* with a reduction in Myc and cyclin D3 levels, and a clear antileukemic effect *in vivo* in patient‐derived xenografts (NOD/SCID mice). *AS1842856* administration was well tolerated by the animals [129]. Another study testing *AS1842856* in B‐ALL showed similar antileukemic effects in both *in vitro* and *in vivo* experiments and confirmed FoxO1 involvement in genome instability tolerance. Notably, increased survival of both PDX mice and mice transplanted with ALL cell lines was achieved with a lower *AS1842856* dose than in the previous study (10 and 30 mg·kg^−1^·day^−1^, respectively, vs. 50 mg·kg^−1^·day^−1^ used in Wang *et al*. [129]). Of note, the study revealed a dose‐dependent reduction in normal pre‐B cells in the bone marrow of heathy mice during toxicity assessment [130].

FoxO1 inhibition in mature B cell malignancies was first tested in BL. Here FoxO1 was found to drive BL cells' clonal expansion, resembling the physiological program of GCs (Fig. 5) [8, 105]. A comprehensive *in vitro* study demonstrated a strong dependency of BL cells on FoxO1 activity level, with both knockdown and inhibition (*AS1842856*; dosed at 40–80 nm) negatively impacting malignant B cell growth [106]. The cytotoxic and antiproliferative effect of FoxO1 inhibition likely stems from the reduction of Myb levels *via miR‐150* induction, a known regulator of Myb [131]. However, the research has not progressed towards any *in vivo* testing so far [106].

In MCL, FoxO1 serves as a master factor driving MCL‐lineage‐specific transcription programs that ensure the survival and proliferation of lymphoma cells (Fig. 5) [9], and a novel FoxO1 inhibitor has been tested in this context (*cpd10*) [123]. Firstly, Jang *et al*. tested the novel FoxO1 inhibitor cpd10 to describe its inhibitory properties (dosed at 2 μm *in vitro* and 100 mg·kg^−1^·day^−1^ *in vivo*) claiming that *cpd10* specifically inhibits FoxO1 activity in luciferase reporter assay with IC_50_ < 0.1 μm. Interestingly, *cpd10* exhibited major differences compared to *AS1842856*. Acutely, treating MCL cell lines with *cpd10* did not interfere with DNA binding, but rather disrupted FoxO1 coactivation by p300 histone acetyltransferase. Prolonged exposure to *cpd10* then led to FoxO1 degradation in MCL cells. The effects of inhibition and genetic *FoxO1* knockout were then compared *via* transcriptomic profiling, showing a large degree of similarity. This suggests a high specificity of the *cpd10* inhibitor. Finally, *cpd10* administration suppressed MCL growth *in vitro* and lymphoma progression *in vivo* (CCMCL1 cell line transplant into NSG mice) with a confirmed effect on *EBF1*, *IRF4*, *PAX5*, *CXCR4*, and *CD79B* mRNA levels. In addition, no apparent toxicity was observed in animals exposed to *cpd10* for over a month [9].

Another piece of evidence supporting FoxO1‐targeted therapy comes from the field of chronic lymphocytic leukemia. As discussed above, CLL cells utilize FoxO1 primarily to maintain ‘tonic’ Akt signaling. FoxO1 also participates in the response to BCR inhibitors, where its transcriptional activity helps CLL cells to adapt to these drugs [10, 108, 112]. Treating CLL cells *in vitro* with *AS1842856* (0.5 μm), alone and in combination with BTK or PI3K inhibitors, led to a high degree of apoptosis and also limited leukemia growth in a xenograft mouse model (30 mg·kg^−1^·day^−1^). Moreover, FoxO1 inhibition impairs primary CLL cell proliferation induced in a co‐culture model of CLL‐T cell interactions [10, 132]. This might be linked not only to Akt signaling but also to FoxO1's role in cell metabolism during pro‐proliferative anabolic changes; however, this will require further investigation.

Outside hematology, a pilot *in vitro* study on various glioblastoma and basal‐like breast carcinoma cell lines demonstrated the potential of FoxO1 inhibition. FoxO1 helps maintain stem‐like gene expression through inducing SOX2 and OCT4 transcription factors (Fig. 5) [133, 134]. Testing both *AS1842856* and *AS1708727* (100 nm to 1 μm) resulted in reduced colony formation ability along with increased expression of pro‐apoptotic proteins BIM and Fas, leading to increased apoptosis [135]. Although FoxO1's participation in stemness maintenance is known, very little has been described about how FoxO1 targets affect cell viability and tumor growth in the context of solid tumors.

Overall, FoxO1 inhibition is emerging as a strategy in cancer therapy, with various preclinical studies demonstrating its potential across different malignancies. In AML and BCP‐ALL, FoxO1 inhibition has shown notable efficacy in reducing cancer stem cell proliferation and cell viability with limited toxicity to healthy cells [124, 129, 130]. Studies in BL, CLL, and MCL have also confirmed FoxO1's role in promoting clonal expansion and cell survival, with both *AS1842856* and *cpd10* proving effective *in vitro* and *in vivo* [9, 10, 106, 108]. Further research should investigate the best strategy to inhibit FoxO1. A key difference between ‘older’ FoxO1 inhibitors like *AS1842856* and the novel *cpd10* is the mechanism of action. While *AS1842856* and *AS1708727* primarily inhibit FoxO1's DNA‐binding activity, *cpd10* disrupts FoxO1's coactivator interactions, particularly with the p300 histone acetyltransferase, and eventually causes FoxO1 degradation [9, 12, 13, 123]. Both *AS1842856* and *cpd10* have demonstrated high efficacy in both *in vitro* and *in vivo* models. However, *cpd10* has less toxicity in rodents over prolonged exposure, suggesting it may be potentially safer (decreased numbers of healthy pre‐B cells in *AS1842856*‐treated mice vs. *cpd10*‐treated mice [9, 130]). A major question is also the specificity of available FoxO1 inhibitors. A direct comparison of *AS1842856* and *cpd10* was performed in a murine diabetic db/db model, showing the superiority of *cpd10* over *AS1842856* in terms of FoxO1‐dependent effects with substantial off‐target activity of *AS1842856* [136]. Transcriptomic profiling of *FoxO1*‐knockout MCL cells revealed a large overlap with the transcriptome of *cdp10*‐treated cells [9], supporting the specificity of this inhibitor. Given the structural similarity between FoxO family members, the specificity of inhibition will be a crucial factor in future applications.

## Conclusions and perspectives

FoxO transcription factors have been fundamentally considered tumor suppressors, since FoxO1, FoxO3a, and FoxO4 triple knockout mice display a strong cancer‐prone phenotype, with lymphomas and hemangiomas being the most prominent malignancies [1]. While FoxO3a rarely acts against this assumption, FoxO1 has been proven to have oncogenic properties in myeloid and B cell malignancies, where FoxO1 inhibition negatively impacts cell fitness. In this regard, FoxO1 can be considered a homeostatic transcription factor, and its context‐specific behavior requires a tightly regulated activity. It seems that cells require ‘just right’ levels of active FoxO1 to optimize their fitness based on specific conditions, as evidenced in pre‐B cell ALL and Burkitt lymphoma [106, 116].

Targeting transcription factors represents a promising yet challenging goal since transcription factors are generally considered hardly druggable due to common intrinsically disordered regions lacking appropriate binding pockets for small‐molecule inhibitors. Various approaches to tackle this were introduced for different transcription factors, such as inhibiting protein interaction (e.g., MDM2‐p53), proteolysis targeting chimeras—PROTACs (e.g., BRD4 and its PROTAC dBET1), modulating upstream regulators (e.g., RUNX1 inhibition *via* CDK7 inhibition) and others [137]. In the case of FoxO1, two separate inhibition mechanisms were developed: inhibiting interaction with DNA (*AS1842856*, *AS1708727*) and inhibiting co‐activating interactions (*cpd10*) [9, 12, 13]. Alternatively, in AML, the concept of blocking FoxO‐specific DNA elements was described [128].

An important feature of FoxO1, which also represents an obstacle for using FoxO1‐targeted therapy, is this transcription factor's extraordinary tissue‐ and context‐specific behavior. In this review, special focus was given to B cells and related malignancies; however, FoxO1 involvement has been described in a vast spectrum of cell processes, including glucose and lipid metabolism [138, 139], stem cell maintenance, differentiation in pancreatic β cells or adipose tissue [124, 140, 141], oxidative stress tolerance and autophagy control in cardiomyocytes [142], and many others that have been reviewed elsewhere [143, 144, 145]. Testing on healthy control mice described an encouragingly good tolerability to long‐term FoxO1 inhibition [9, 129, 130]; however, testing in large animals remains to be performed before phase I studies in humans. Furthermore, two patent applications (US10544415B2, US20230416228A1) in the field of diabetes focused on the induction of insulin‐producing gut β‐like cells *via* FoxO1 inhibition; studies in diabetes currently represent the closest point towards clinical testing of FoxO1 inhibitors [146, 147, 148].

In B cell malignancies, the attraction of FoxO1 as a target is further underscored by the observation that FoxO1 represses transcription of CD20 cell‐surface B cell marker (encoded by *MS4A1*), namely in CLL and DLBCL [10, 108, 149, 150]. Anti‐CD20 monoclonal antibodies (e.g., rituximab, obinutuzumab) are frequently used in treating B cell neoplasms together with chemotherapy or small‐molecule inhibitors [149]. In line with FoxO1 repressing CD20 transcription, activating *FoxO1* mutations correlate with decreased overall survival of DLBCL patients treated with anti‐CD20‐based therapy [11, 150]. Moreover, relapsed DLBCL samples had an increased frequency of *FoxO1* mutations (8.6% in *de novo* DLBCL vs. ~ 27% at relapse) [151]. Morin and colleagues showed that both T24A (activating) and S205N (transcriptionally inactivating) *FoxO1* mutations clonally expand during relapse following R‐CHOP therapy [151]. These data support the rationale for combining FoxO1 inhibition with anti‐CD20 antibodies, which remain a cornerstone of both chemo‐based and chemo‐free regimens. On the contrary, FoxO1 inhibition might limit the efficacy of chimeric antigen receptor (CAR) T cell therapy. CAR T cells' exhaustion and differentiation are major factors reducing their cytotoxicity and anti‐tumor effects [152, 153]. FoxO1 has been recently described as a key cell factor maintaining metabolic fitness and a ‘stemness‐like’ phenotype that both significantly enhance CAR T cells' cytotoxic capacity/durability. FoxO1 activity also highly correlates with clinical responses to CAR T cells in CLL and ALL [154, 155].

In summary, the FoxO1 transcription factor was previously considered to be a protein with an exclusive tumor‐suppressive role. However, in the last decade, a number of studies have revealed that FoxO1's involvement in directing cell fate is extraordinarily complex, and FoxO1 can be crucial in supporting the fitness of malignant B cells and many other cell types. FoxO1's bivalent behavior may result in contradictory observations, where for example, both high and low FoxO1 activity negatively impact the survival of the same malignant cells. Cell fitness seems to increase when the FoxO1 activity is ‘just right’ and this balance might shift during (patho)physiological processes such as proliferation or increased metabolic output. We expect that studies of FoxO1 biology and inhibition will potentially lead to novel therapies and rational therapeutic combinations in oncology.

## Author contributions

KH and PP contributed equally to the manuscript writing. LO and MM revised and edited the manuscript.

## References

[1] Paik J‐H , Kollipara R , Chu G , Ji H , Xiao Y , Ding Z , Miao L , Tothova Z , Horner JW , Carrasco DR *et al*. (2007) FoxOs are lineage‐restricted redundant tumor suppressors and critical regulators of endothelial cell homeostasis. Cell 128, 309–323. 17254969 10.1016/j.cell.2006.12.029 PMC1855089

[2] Dengler HS , Baracho GV , Omori SA , Bruckner S , Arden K , Castrillon DH , DePinho RA and Rickert RC (2008) Distinct roles for Foxo1 at multiple stages of B cell differentiation. Nat Immunol 9, 1388–1398. 18978794 10.1038/ni.1667 PMC2679692

[3] Srinivasan L , Sasaki Y , Calado DP , Zhang B , Paik JH , DePinho RA , Kutok JL , Kearney JF , Otipoby KL and Rajewsky K (2009) PI3 kinase signals BCR‐dependent mature B cell survival. Cell 139, 573–586. 19879843 10.1016/j.cell.2009.08.041 PMC2787092

[4] Dominguez‐Sola D , Kung J , Holmes AB , Wells VA , Mo T , Basso K and Dalla‐Favera R (2015) The FOXO1 transcription factor instructs the germinal center dark zone program. Immunity 43, 1064–1074. 26620759 10.1016/j.immuni.2015.10.015

[5] Sander S , Chu VT , Yasuda T , Franklin A , Graf R , Calado DP , Li S , Imami K , Selbach M , Di Virgilio M *et al*. (2015) PI3 kinase and FOXO1 transcription factor activity differentially control B cells in the germinal center light and dark zones. Immunity 43, 1075–1086. 26620760 10.1016/j.immuni.2015.10.021

[6] Xie L , Ushmorov A , Leithäuser F , Guan H , Steidl C , Färbinger J , Pelzer C , Vogel MJ , Maier HJ , Gascoyne RD *et al*. (2012) FOXO1 is a tumor suppressor in classical Hodgkin lymphoma. Blood 119, 3503–3511. 22343918 10.1182/blood-2011-09-381905

[7] Bloedjes TA , De Wilde G , Maas C , Eldering E , Bende RJ , Van Noesel CJM , Pals ST , Spaargaren M and Guikema JEJ (2020) AKT signaling restrains tumor suppressive functions of FOXO transcription factors and GSK3 kinase in multiple myeloma. Blood Adv 4, 4151–4164. 32898245 10.1182/bloodadvances.2019001393 PMC7479958

[8] Zhou P , Blain AE , Newman AM , Zaka M , Chagaluka G , Adlar FR , Offor UT , Broadbent C , Chaytor L , Whitehead A *et al*. (2019) Sporadic and endemic Burkitt lymphoma have frequent FOXO1 mutations but distinct hotspots in the AKT recognition motif. Blood Adv 3, 2118–2127. 31300419 10.1182/bloodadvances.2018029546 PMC6650741

[9] Jang J‐Y , Hwang I , Pan H , Yao J , Alinari L , Imada E , Zanettini C , Kluk MJ , Wang Y , Lee Y *et al*. (2022) A FOXO1‐dependent transcription network is a targetable vulnerability of mantle cell lymphomas. J Clin Invest 132, e160767. 36282572 10.1172/JCI160767 PMC9753996

[10] Ondrisova L , Seda V , Hlavac K , Pavelkova P , Hoferkova E , Chiodin G , Kostalova L , Mladonicka Pavlasova G , Filip D , Vecera J *et al*. (2024) FoxO1/Rictor axis induces a non‐genetic adaptation to Ibrutinib via Akt activation in chronic lymphocytic leukemia. J Clin Invest e173770. doi: 10.1172/JCI173770 39436708 PMC11601945

[11] Trinh DL , Scott DW , Morin RD , Mendez‐Lago M , An J , Jones SJM , Mungall AJ , Zhao Y , Schein J , Steidl C *et al*. (2013) Analysis of FOXO1 mutations in diffuse large B‐cell lymphoma. Blood 121, 3666–3674. 23460611 10.1182/blood-2013-01-479865 PMC3643765

[12] Nagashima T , Shigematsu N , Maruki R , Urano Y , Tanaka H , Shimaya A , Shimokawa T and Shibasaki M (2010) Discovery of novel Forkhead box O1 inhibitors for treating type 2 diabetes: improvement of fasting Glycemia in diabetic *db/db* mice. Mol Pharmacol 78, 961–970. 20736318 10.1124/mol.110.065714

[13] Tanaka H , Nagashima T , Shimaya A , Urano Y , Shimokawa T and Shibasaki M (2010) Effects of the novel Foxo1 inhibitor AS1708727 on plasma glucose and triglyceride levels in diabetic db/db mice. Eur J Pharmacol 645, 185–191. 20655898 10.1016/j.ejphar.2010.07.018

[14] Brent MM , Anand R and Marmorstein R (2008) Structural basis for DNA recognition by FoxO1 and its regulation by posttranslational modification. Structure 16, 1407–1416. 18786403 10.1016/j.str.2008.06.013 PMC2597217

[15] Psenakova K , Kohoutova K , Obsilova V , Ausserlechner MJ , Veverka V and Obsil T (2019) Forkhead domains of FOXO transcription factors differ in both overall conformation and dynamics. Cells 8, E966. 10.3390/cells8090966 PMC6770010 31450545

[16] Hatta M and Cirillo LA (2007) Chromatin opening and stable perturbation of core histone:DNA contacts by FoxO1. J Biol Chem 282, 35583–35593. 17923482 10.1074/jbc.M704735200

[17] Wang F , Marshall CB , Yamamoto K , Li G‐Y , Plevin MJ , You H , Mak TW and Ikura M (2008) Biochemical and structural characterization of an intramolecular interaction in FOXO3a and its binding with p53. J Mol Biol 384, 590–603. 18824006 10.1016/j.jmb.2008.09.025

[18] Obsil T and Obsilova V (2008) Structure/function relationships underlying regulation of FOXO transcription factors. Oncogene 27, 2263–2275. 18391969 10.1038/onc.2008.20

[19] Jacobs FMJ , van der Heide LP , Wijchers PJEC , Burbach JPH , Hoekman MFM and Smidt MP (2003) FoxO6, a novel member of the FoxO class of transcription factors with distinct shuttling dynamics. J Biol Chem 278, 35959–35967. 12857750 10.1074/jbc.M302804200

[20] Lees J , Hay J , Moles MW and Michie AM (2023) The discrete roles of individual FOXO transcription factor family members in B‐cell malignancies. Front Immunol 14, 1179101. 37275916 10.3389/fimmu.2023.1179101 PMC10233034

[21] Oh H‐M , Yu C‐R , Golestaneh N , Amadi‐Obi A , Lee YS , Eseonu A , Mahdi RM and Egwuagu CE (2011) STAT3 protein promotes T‐cell survival and inhibits interleukin‐2 production through up‐regulation of class O Forkhead transcription factors. J Biol Chem 286, 30888–30897. 21730069 10.1074/jbc.M111.253500 PMC3162449

[22] Welinder E , Mansson R , Mercer EM , Bryder D , Sigvardsson M and Murre C (2011) The transcription factors E2A and HEB act in concert to induce the expression of FOXO1 in the common lymphoid progenitor. Proc Natl Acad Sci U S A 108, 17402–17407. 21972416 10.1073/pnas.1111766108 PMC3198373

[23] Essaghir A , Dif N , Marbehant CY , Coffer PJ and Demoulin J‐B (2009) The transcription of FOXO genes is stimulated by FOXO3 and repressed by growth factors. J Biol Chem 284, 10334–10342. 19244250 10.1074/jbc.M808848200 PMC2667720

[24] Gaudette BT , Dwivedi B , Chitta KS , Poulain S , Powell D , Vertino P , Leleu X , Lonial S , Chanan‐Khan AA , Kowalski J *et al*. (2016) Low expression of pro‐apoptotic Bcl‐2 family proteins sets the apoptotic threshold in Waldenström macroglobulinemia. Oncogene 35, 479–490. 25893290 10.1038/onc.2015.103 PMC4874246

[25] Osswald CD , Xie L , Guan H , Herrmann F , Pick SM , Vogel MJ , Gehringer F , Chan FC , Steidl C , Wirth T *et al*. (2018) Fine‐tuning of FOXO3A in cHL as a survival mechanism and a hallmark of abortive plasma cell differentiation. Blood 131, 1556–1567. 29439954 10.1182/blood-2017-07-795278 PMC5887767

[26] Dobson M , Ramakrishnan G , Ma S , Kaplun L , Balan V , Fridman R and Tzivion G (2011) Bimodal regulation of FoxO3 by AKT and 14‐3‐3. Biochim Biophys Acta 1813, 1453–1464. 21621563 10.1016/j.bbamcr.2011.05.001 PMC3237389

[27] Ushmorov A and Wirth T (2018) FOXO in B‐cell lymphopoiesis and B cell neoplasia. Semin Cancer Biol 50, 132–141. 28774833 10.1016/j.semcancer.2017.07.008

[28] Brownawell AM , Kops GJ , Macara IG and Burgering BM (2001) Inhibition of nuclear import by protein kinase B (Akt) regulates the subcellular distribution and activity of the forkhead transcription factor AFX. Mol Cell Biol 21, 3534–3546. 11313479 10.1128/MCB.21.10.3534-3546.2001 PMC100275

[29] Brunet A , Bonni A , Zigmond MJ , Lin MZ , Juo P , Hu LS , Anderson MJ , Arden KC , Blenis J and Greenberg ME (1999) Akt promotes cell survival by phosphorylating and inhibiting a Forkhead transcription factor. Cell 96, 857–868. 10102273 10.1016/s0092-8674(00)80595-4

[30] Guo S , Rena G , Cichy S , He X , Cohen P and Unterman T (1999) Phosphorylation of serine 256 by protein kinase B disrupts transactivation by FKHR and mediates effects of insulin on insulin‐like growth factor‐binding protein‐1 promoter activity through a conserved insulin response sequence. J Biol Chem 274, 17184–17192. 10358076 10.1074/jbc.274.24.17184

[31] Nielsen MD , Luo X , Biteau B , Syverson K and Jasper H (2008) 14‐3‐3ε antagonizes FoxO to control growth, apoptosis and longevity in drosophila. Aging Cell 7, 688–699. 18665908 10.1111/j.1474-9726.2008.00420.x PMC3851013

[32] Obsilova V , Vecer J , Herman P , Pabianova A , Sulc M , Teisinger J , Boura E and Obsil T (2005) 14‐3‐3 protein interacts with nuclear localization sequence of Forkhead transcription factor FoxO4. Biochemistry 44, 11608–11617. 16114898 10.1021/bi050618r

[33] Silhan J , Vacha P , Strnadova P , Vecer J , Herman P , Sulc M , Teisinger J , Obsilova V and Obsil T (2009) 14‐3‐3 protein masks the DNA binding interface of forkhead transcription factor FOXO4. J Biol Chem 284, 19349–19360. 19416966 10.1074/jbc.M109.002725 PMC2740560

[34] Christensen R , de la Torre‐Ubieta L , Bonni A and Colón‐Ramos DA (2011) A conserved PTEN/FOXO pathway regulates neuronal morphology during *C. elegans* development. Development 138, 5257–5267. 22069193 10.1242/dev.069062 PMC3210501

[35] Yan L , Lavin VA , Moser LR , Cui Q , Kanies C and Yang E (2008) PP2A regulates the pro‐apoptotic activity of FOXO1. J Biol Chem 283, 7411–7420. 18211894 10.1074/jbc.M708083200 PMC2276329

[36] Brenkman AB , de Keizer PLJ , van den Broek NJF , Jochemsen AG and Burgering BMT (2008) Mdm2 induces mono‐ubiquitination of FOXO4. PLoS One 3, e2819. 18665269 10.1371/journal.pone.0002819 PMC2475507

[37] Matsuzaki H , Daitoku H , Hatta M , Aoyama H , Yoshimochi K and Fukamizu A (2005) Acetylation of Foxo1 alters its DNA‐binding ability and sensitivity to phosphorylation. Proc Natl Acad Sci U S A 102, 11278–11283. 16076959 10.1073/pnas.0502738102 PMC1183558

[38] Yamagata K , Daitoku H , Takahashi Y , Namiki K , Hisatake K , Kako K , Mukai H , Kasuya Y and Fukamizu A (2008) Arginine methylation of FOXO transcription factors inhibits their phosphorylation by Akt. Mol Cell 32, 221–231. 18951090 10.1016/j.molcel.2008.09.013

[39] Ramaswamy S , Nakamura N , Sansal I , Bergeron L and Sellers WR (2002) A novel mechanism of gene regulation and tumor suppression by the transcription factor FKHR. Cancer Cell 2, 81–91. 12150827 10.1016/s1535-6108(02)00086-7

[40] Schmidt M , Fernandez de Mattos S , van der Horst A , Klompmaker R , Kops GJPL , Lam EW‐F , Burgering BMT and Medema RH (2002) Cell cycle inhibition by FoxO forkhead transcription factors involves downregulation of cyclin D. Mol Cell Biol 22, 7842–7852. 12391153 10.1128/MCB.22.22.7842-7852.2002 PMC134724

[41] Nakamura N , Ramaswamy S , Vazquez F , Signoretti S , Loda M and Sellers WR (2000) Forkhead transcription factors are critical effectors of cell death and cell cycle arrest downstream of PTEN. Mol Cell Biol 20, 8969–8982. 11073996 10.1128/mcb.20.23.8969-8982.2000 PMC86551

[42] Tran H , Brunet A , Grenier JM , Datta SR , Fornace AJ , DiStefano PS , Chiang LW and Greenberg ME (2002) DNA repair pathway stimulated by the forkhead transcription factor FOXO3a through the Gadd45 protein. Science 296, 530–534. 11964479 10.1126/science.1068712

[43] Yalcin S , Zhang X , Luciano JP , Mungamuri SK , Marinkovic D , Vercherat C , Sarkar A , Grisotto M , Taneja R and Ghaffari S (2008) Foxo3 is essential for the regulation of ataxia telangiectasia mutated and oxidative stress‐mediated homeostasis of hematopoietic stem cells. J Biol Chem 283, 25692–25705. 18424439 10.1074/jbc.M800517200

[44] Kops GJPL , Dansen TB , Polderman PE , Saarloos I , Wirtz KWA , Coffer PJ , Huang T‐T , Bos JL , Medema RH and Burgering BMT (2002) Forkhead transcription factor FOXO3a protects quiescent cells from oxidative stress. Nature 419, 316–321. 12239572 10.1038/nature01036

[45] Modur V , Nagarajan R , Evers BM and Milbrandt J (2002) FOXO proteins regulate tumor necrosis factor‐related apoptosis inducing ligand expression. Implications for PTEN mutation in prostate cancer. J Biol Chem 277, 47928–47937. 12351634 10.1074/jbc.M207509200

[46] Stahl M , Dijkers PF , Kops GJPL , Lens SMA , Coffer PJ , Burgering BMT and Medema RH (2002) The Forkhead transcription factor FoxO regulates transcription of p27 *Kip1* and Bim in response to IL‐2. J Immunol 168, 5024–5031. 11994454 10.4049/jimmunol.168.10.5024

[47] You H , Pellegrini M , Tsuchihara K , Yamamoto K , Hacker G , Erlacher M , Villunger A and Mak TW (2006) FOXO3a‐dependent regulation of Puma in response to cytokine/growth factor withdrawal. J Exp Med 203, 1657–1663. 16801400 10.1084/jem.20060353 PMC2118330

[48] Huang H , Regan KM , Lou Z , Chen J and Tindall DJ (2006) CDK2‐dependent phosphorylation of FOXO1 as an apoptotic response to DNA damage. Science 314, 294–297. 17038621 10.1126/science.1130512

[49] Rena G , Guo S , Cichy SC , Unterman TG and Cohen P (1999) Phosphorylation of the transcription factor Forkhead family member FKHR by protein kinase B. J Biol Chem 274, 17179–17183. 10358075 10.1074/jbc.274.24.17179

[50] Bashir T , Dorrello NV , Amador V , Guardavaccaro D and Pagano M (2004) Control of the SCF(Skp2‐Cks1) ubiquitin ligase by the APC/C(Cdh1) ubiquitin ligase. Nature 428, 190–193. 15014502 10.1038/nature02330

[51] Wei W , Ayad NG , Wan Y , Zhang G‐J , Kirschner MW and Kaelin WG (2004) Degradation of the SCF component Skp2 in cell‐cycle phase G1 by the anaphase‐promoting complex. Nature 428, 194–198. 15014503 10.1038/nature02381

[52] Liu P , Kao TP and Huang H (2008) CDK1 promotes cell proliferation and survival via phosphorylation and inhibition of FOXO1 transcription factor. Oncogene 27, 4733–4744. 18408765 10.1038/onc.2008.104

[53] Cheng M , Nie Y , Song M , Chen F and Yu Y (2024) Forkhead box O proteins: steering the course of stem cell fate. Cell Regen 13, 7. 38466341 10.1186/s13619-024-00190-1 PMC10928065

[54] Renault VM , Rafalski VA , Morgan AA , Salih DAM , Brett JO , Webb AE , Villeda SA , Thekkat PU , Guillerey C , Denko NC *et al*. (2009) FoxO3 regulates neural stem cell homeostasis. Cell Stem Cell 5, 527–539. 19896443 10.1016/j.stem.2009.09.014 PMC2775802

[55] García‐Prat L , Perdiguero E , Alonso‐Martín S , Dell'Orso S , Ravichandran S , Brooks SR , Juan AH , Campanario S , Jiang K , Hong X *et al*. (2020) FoxO maintains a genuine muscle stem‐cell quiescent state until geriatric age. Nat Cell Biol 22, 1307–1318. 33106654 10.1038/s41556-020-00593-7

[56] Webb AE , Pollina EA , Vierbuchen T , Urbán N , Ucar D , Leeman DS , Martynoga B , Sewak M , Rando TA , Guillemot F *et al*. (2013) FOXO3 shares common targets with ASCL1 genome‐wide and inhibits ASCL1‐dependent neurogenesis. Cell Rep 4, 477–491. 23891001 10.1016/j.celrep.2013.06.035 PMC3838667

[57] Audesse AJ , Dhakal S , Hassell L‐A , Gardell Z , Nemtsova Y and Webb AE (2019) FOXO3 directly regulates an autophagy network to functionally regulate proteostasis in adult neural stem cells. PLoS Genet 15, e1008097. 30973875 10.1371/journal.pgen.1008097 PMC6478346

[58] Warr MR , Binnewies M , Flach J , Reynaud D , Garg T , Malhotra R , Debnath J and Passegué E (2013) FOXO3A directs a protective autophagy program in haematopoietic stem cells. Nature 494, 323–327. 23389440 10.1038/nature11895 PMC3579002

[59] Zhang X , Yalcin S , Lee D‐F , Yeh T‐YJ , Lee S‐M , Su J , Mungamuri SK , Rimmelé P , Kennedy M , Sellers R *et al*. (2011) FOXO1 is an essential regulator of pluripotency in human embryonic stem cells. Nat Cell Biol 13, 1092–1099. 21804543 10.1038/ncb2293 PMC4053529

[60] Liu P , Liu K , Gu H , Wang W , Gong J , Zhu Y , Zhao Q , Cao J , Han C , Gao F *et al*. (2017) High autophagic flux guards ESC identity through coordinating autophagy machinery gene program by FOXO1. Cell Death Differ 24, 1672–1680. 28622295 10.1038/cdd.2017.90 PMC5596427

[61] Vilchez D , Boyer L , Morantte I , Lutz M , Merkwirth C , Joyce D , Spencer B , Page L , Masliah E , Berggren WT *et al*. (2012) Increased proteasome activity in human embryonic stem cells is regulated by PSMD11. Nature 489, 304–308. 22972301 10.1038/nature11468 PMC5215918

[62] Bakker WJ , Blázquez‐Domingo M , Kolbus A , Besooyen J , Steinlein P , Beug H , Coffer PJ , Löwenberg B , Von Lindern M and Van Dijk TB (2004) FoxO3a regulates erythroid differentiation and induces BTG1, an activator of protein arginine methyl transferase 1. J Cell Biol 164, 175–184. 14734530 10.1083/jcb.200307056 PMC2172323

[63] Teixeira CC , Liu Y , Thant LM , Pang J , Palmer G and Alikhani M (2010) Foxo1, a novel regulator of osteoblast differentiation and Skeletogenesis. J Biol Chem 285, 31055–31065. 20650891 10.1074/jbc.M109.079962 PMC2945596

[64] Iyer S , Ambrogini E , Bartell SM , Han L , Roberson PK , De Cabo R , Jilka RL , Weinstein RS , O'Brien CA , Manolagas SC *et al*. (2013) FOXOs attenuate bone formation by suppressing Wnt signaling. J Clin Invest 123, 3409–3419. 23867625 10.1172/JCI68049 PMC3726166

[65] Higuchi M , Dusting GJ , Peshavariya H , Jiang F , Hsiao ST‐F , Chan EC and Liu G‐S (2013) Differentiation of human adipose‐derived stem cells into fat involves reactive oxygen species and Forkhead box O1 mediated upregulation of antioxidant enzymes. Stem Cells Dev 22, 878–888. 23025577 10.1089/scd.2012.0306 PMC3585477

[66] Kurakazu I , Akasaki Y , Hayashida M , Tsushima H , Goto N , Sueishi T , Toya M , Kuwahara M , Okazaki K , Duffy T *et al*. (2019) FOXO1 transcription factor regulates chondrogenic differentiation through transforming growth factor β1 signaling. J Biol Chem 294, 17555–17569. 31601652 10.1074/jbc.RA119.009409 PMC6873195

[67] Yuan S , Zhang L , Ji L , Zhong S , Jiang L , Wan Y , Song Y , Zhang C and Wang R (2022) FoxO3a cooperates with RUNX1 to promote chondrogenesis and terminal hypertrophic of the chondrogenic progenitor cells. Biochem Biophys Res Commun 589, 41–47. 34891040 10.1016/j.bbrc.2021.12.008

[68] Miyamoto K , Miyamoto T , Kato R , Yoshimura A , Motoyama N and Suda T (2008) FoxO3a regulates hematopoietic homeostasis through a negative feedback pathway in conditions of stress or aging. Blood 112, 4485–4493. 18799725 10.1182/blood-2008-05-159848 PMC2954681

[69] Tsai W‐B , Chung YM , Takahashi Y , Xu Z and Hu MC‐T (2008) Functional interaction between FOXO3a and ATM regulates DNA damage response. Nat Cell Biol 10, 460–467. 18344987 10.1038/ncb1709 PMC2674111

[70] Peña‐Pérez L , Kharazi S , Frengen N , Krstic A , Bouderlique T , Hauenstein J , He M , Somuncular E , Li Wang X , Dahlberg C *et al*. (2022) FOXO dictates initiation of B cell development and myeloid restriction in common lymphoid progenitors. Front Immunol 13, 880668. 35603175 10.3389/fimmu.2022.880668 PMC9116193

[71] Kuo TC and Schlissel MS (2009) Mechanisms controlling expression of the RAG locus during lymphocyte development. Curr Opin Immunol 21, 173–178. 19359154 10.1016/j.coi.2009.03.008 PMC2676217

[72] Amin RH and Schlissel MS (2008) Foxo1 directly regulates the transcription of recombination‐activating genes during B cell development. Nat Immunol 9, 613–622. 18469817 10.1038/ni.1612 PMC2612116

[73] Lam K‐P , Kühn R and Rajewsky K (1997) In vivo ablation of surface immunoglobulin on mature B cells by inducible gene targeting results in rapid cell death. Cell 90, 1073–1083. 9323135 10.1016/s0092-8674(00)80373-6

[74] Lee K , Heffington L , Jellusova J , Nam KT , Raybuck A , Cho SH , Thomas JW , Rickert RC and Boothby M (2013) Requirement for Rictor in homeostasis and function of mature B lymphoid cells. Blood 122, 2369–2379. 23958952 10.1182/blood-2013-01-477505 PMC3790507

[75] Yusuf I , Zhu X , Kharas MG , Chen J and Fruman DA (2004) Optimal B‐cell proliferation requires phosphoinositide 3‐kinase‐dependent inactivation of FOXO transcription factors. Blood 104, 784–787. 15069012 10.1182/blood-2003-09-3071

[76] Calamito M , Juntilla MM , Thomas M , Northrup DL , Rathmell J , Birnbaum MJ , Koretzky G and Allman D (2010) Akt1 and Akt2 promote peripheral B‐cell maturation and survival. Blood 115, 4043–4050. 20042722 10.1182/blood-2009-09-241638 PMC2875094

[77] Chen J , Limon JJ , Blanc C , Peng SL and Fruman DA (2010) Foxo1 regulates marginal zone B‐cell development. Eur J Immunol 40, 1890–1896. 20449867 10.1002/eji.200939817 PMC2926184

[78] Suzuki A , Kaisho T , Ohishi M , Tsukio‐Yamaguchi M , Tsubata T , Koni PA , Sasaki T , Mak TW and Nakano T (2003) Critical roles of Pten in B cell homeostasis and immunoglobulin class switch recombination. J Exp Med 197, 657–667. 12615906 10.1084/jem.20021101 PMC2193827

[79] Cox E‐M , El‐Behi M , Ries S , Vogt JF , Kohlhaas V , Michna T , Manfroi B , Al‐Maarri M , Wanke F , Tirosh B *et al*. (2023) AKT activity orchestrates marginal zone B cell development in mice and humans. Cell Rep 42, 112378. 37060566 10.1016/j.celrep.2023.112378

[80] Setz CS , Khadour A , Renna V , Iype J , Gentner E , He X , Datta M , Young M , Nitschke L , Wienands J *et al*. (2019) Pten controls B‐cell responsiveness and germinal center reaction by regulating the expression of IgD BCR. EMBO J 38, e100249. 31015337 10.15252/embj.2018100249 PMC6545559

[81] Inoue T , Shinnakasu R , Ise W , Kawai C , Egawa T and Kurosaki T (2017) The transcription factor Foxo1 controls germinal center B cell proliferation in response to T cell help. J Exp Med 214, 1181–1198. 28351982 10.1084/jem.20161263 PMC5379976

[82] Oestreich KJ , Mohn SE and Weinmann AS (2012) Molecular mechanisms that control the expression and activity of Bcl‐6 in TH1 cells to regulate flexibility with a TFH‐like gene profile. Nat Immunol 13, 405–411. 22406686 10.1038/ni.2242 PMC3561768

[83] Park S‐R , Zan H , Pal Z , Zhang J , Al‐Qahtani A , Pone EJ , Xu Z , Mai T and Casali P (2009) HoxC4 binds to the promoter of the cytidine deaminase AID gene to induce AID expression, class‐switch DNA recombination and somatic hypermutation. Nat Immunol 10, 540–550. 19363484 10.1038/ni.1725 PMC2753990

[84] Omori SA , Cato MH , Anzelon‐Mills A , Puri KD , Shapiro‐Shelef M , Calame K and Rickert RC (2006) Regulation of class‐switch recombination and plasma cell differentiation by phosphatidylinositol 3‐kinase signaling. Immunity 25, 545–557. 17000121 10.1016/j.immuni.2006.08.015

[85] Roberto MP , Varano G , Vinas‐Castells R , Holmes AB , Kumar R , Pasqualucci L , Farinha P , Scott DW and Dominguez‐Sola D (2021) Mutations in the transcription factor FOXO1 mimic positive selection signals to promote germinal center B cell expansion and lymphomagenesis. Immunity 54, 1807–1824.e14. 34380064 10.1016/j.immuni.2021.07.009 PMC8475267

[86] Cornelis R , Hahne S , Taddeo A , Petkau G , Malko D , Durek P , Thiem M , Heiberger L , Peter L , Mohr E *et al*. (2020) Stromal cell‐contact dependent PI3K and APRIL induced NF‐κB signaling prevent mitochondrial‐ and ER stress induced death of memory plasma cells. Cell Rep 32, 107982. 32755576 10.1016/j.celrep.2020.107982 PMC7408492

[87] Vogel MJ , Xie L , Guan H , Tooze RM , Maier T , Kostezka U , Maier HJ , Holzmann K , Chan FC , Steidl C *et al*. (2014) FOXO1 repression contributes to block of plasma cell differentiation in classical Hodgkin lymphoma. Blood 124, 3118–3129. 25232062 10.1182/blood-2014-07-590570

[88] Xie L , Ritz O , Leithäuser F , Guan H , Färbinger J , Weitzer CD , Gehringer F , Brüderlein S , Holzmann K , Vogel MJ *et al*. (2014) FOXO1 downregulation contributes to the oncogenic program of primary mediastinal B‐cell lymphoma. Oncotarget 5, 5392–5402. 24977668 10.18632/oncotarget.2107 PMC4170625

[89] Rizk M , Rizq O , Oshima M , Nakajima‐Takagi Y , Koide S , Saraya A , Isshiki Y , Chiba T , Yamazaki S , Ma A *et al*. (2019) Akt inhibition synergizes with polycomb repressive complex 2 inhibition in the treatment of multiple myeloma. Cancer Sci 110, 3695–3707. 31571328 10.1111/cas.14207 PMC6890440

[90] Corno C , Stucchi S , De Cesare M , Carenini N , Stamatakos S , Ciusani E , Minoli L , Scanziani E , Argueta C , Landesman Y *et al*. (2018) FoxO‐1 contributes to the efficacy of the combination of the XPO1 inhibitor selinexor and cisplatin in ovarian carcinoma preclinical models. Biochem Pharmacol 147, 93–103. 29155058 10.1016/j.bcp.2017.11.009

[91] Wang J , Sun T , Meng Z , Wang L , Li M , Chen J , Qin T , Yu J , Zhang M , Bie Z *et al*. (2021) XPO1 inhibition synergizes with PARP1 inhibition in small cell lung cancer by targeting nuclear transport of FOXO3a. Cancer Lett 503, 197–212. 33493586 10.1016/j.canlet.2021.01.008

[92] Uddin S , Hussain AR , Siraj AK , Manogaran PS , Al‐Jomah NA , Moorji A , Atizado V , Al‐Dayel F , Belgaumi A , El‐Solh H *et al*. (2006) Role of phosphatidylinositol 3′‐kinase/AKT pathway in diffuse large B‐cell lymphoma survival. Blood 108, 4178–4186. 16946303 10.1182/blood-2006-04-016907

[93] Pfeifer M , Grau M , Lenze D , Wenzel S‐S , Wolf A , Wollert‐Wulf B , Dietze K , Nogai H , Storek B , Madle H *et al*. (2013) PTEN loss defines a PI3K/AKT pathway‐dependent germinal center subtype of diffuse large B‐cell lymphoma. Proc Natl Acad Sci U S A 110, 12420–12425. 23840064 10.1073/pnas.1305656110 PMC3725065

[94] Go H , Jang J‐Y , Kim P‐J , Kim Y‐G , Nam SJ , Paik JH , Kim TM , Heo DS , Kim C‐W and Jeon YK (2015) MicroRNA‐21 plays an oncogenic role by targeting FOXO1 and activating the PI3K/AKT pathway in diffuse large B‐cell lymphoma. Oncotarget 6, 15035–15049. 25909227 10.18632/oncotarget.3729 PMC4558134

[95] Szydlowski M , Kiliszek P , Sewastianik T , Jablonska E , Bialopiotrowicz E , Gorniak P , Polak A , Markowicz S , Nowak E , Grygorowicz MA *et al*. (2016) FOXO1 activation is an effector of SYK and AKT inhibition in tonic BCR signal‐dependent diffuse large B‐cell lymphomas. Blood 127, 739–748. 26585955 10.1182/blood-2015-06-654111

[96] Chen L , Monti S , Juszczynski P , Ouyang J , Chapuy B , Neuberg D , Doench JG , Bogusz AM , Habermann TM , Dogan A *et al*. (2013) SYK inhibition modulates distinct PI3K/AKT‐ dependent survival pathways and cholesterol biosynthesis in diffuse large B cell lymphomas. Cancer Cell 23, 826–838. 23764004 10.1016/j.ccr.2013.05.002 PMC3700321

[97] Sablon A , Bollaert E , Pirson C , Velghe AI and Demoulin J‐B (2022) FOXO1 forkhead domain mutants in B‐cell lymphoma lack transcriptional activity. Sci Rep 12, 1309. 35079069 10.1038/s41598-022-05334-4 PMC8789783

[98] Layden HM , Ellis JD , Bomber ML , Bartlett LN , Hiebert SW and Stengel KR (2024) Mutant FOXO1 controls an oncogenic network via enhancer accessibility. Cell Genomics 4, 100537. 38604128 10.1016/j.xgen.2024.100537 PMC11019358

[99] Ning N , Zhang S , Wu Q , Li X , Kuang D , Duan Y , Xia M , Liu H , Weng J , Ba H *et al*. (2022) Inhibition of acylglycerol kinase sensitizes DLBCL to venetoclax via upregulation of FOXO1‐mediated BCL‐2 expression. Theranostics 12, 5537–5550. 35910796 10.7150/thno.72786 PMC9330532

[100] Devan J , Janikova A and Mraz M (2018) New concepts in follicular lymphoma biology: from BCL2 to epigenetic regulators and non‐coding RNAs. Semin Oncol 45, 291–302. 30360879 10.1053/j.seminoncol.2018.07.005

[101] Phan RT and Dalla‐Favera R (2004) The BCL6 proto‐oncogene suppresses p53 expression in germinal‐centre B cells. Nature 432, 635–639. 15577913 10.1038/nature03147

[102] Pasqualucci L , Khiabanian H , Fangazio M , Vasishtha M , Messina M , Holmes AB , Ouillette P , Trifonov V , Rossi D , Tabbò F *et al*. (2014) Genetics of follicular lymphoma transformation. Cell Rep 6, 130–140. 24388756 10.1016/j.celrep.2013.12.027 PMC4100800

[103] Pastore A , Jurinovic V , Kridel R , Hoster E , Staiger AM , Szczepanowski M , Pott C , Kopp N , Murakami M , Horn H *et al*. (2015) Integration of gene mutations in risk prognostication for patients receiving first‐line immunochemotherapy for follicular lymphoma: a retrospective analysis of a prospective clinical trial and validation in a population‐based registry. Lancet Oncol 16, 1111–1122. 26256760 10.1016/S1470-2045(15)00169-2

[104] Bouska A , Zhang W , Gong Q , Iqbal J , Scuto A , Vose J , Ludvigsen M , Fu K , Weisenburger DD , Greiner TC *et al*. (2017) Combined copy number and mutation analysis identifies oncogenic pathways associated with transformation of follicular lymphoma. Leukemia 31, 83–91. 27389057 10.1038/leu.2016.175 PMC5214175

[105] Kabrani E , Chu VT , Tasouri E , Sommermann T , Baßler K , Ulas T , Zenz T , Bullinger L , Schultze JL , Rajewsky K *et al*. (2018) Nuclear FOXO1 promotes lymphomagenesis in germinal center B cells. Blood 132, 2670–2683. 30333121 10.1182/blood-2018-06-856203

[106] Gehringer F , Weissinger SE , Swier LJ , Möller P , Wirth T and Ushmorov A (2019) FOXO1 confers maintenance of the dark zone proliferation and survival program and can Be pharmacologically targeted in Burkitt lymphoma. Cancer 11, E1427. 10.3390/cancers11101427 PMC6826697 31557894

[107] Cosimo E , Tarafdar A , Moles MW , Holroyd AK , Malik N , Catherwood MA , Hay J , Dunn KM , Macdonald AM , Guichard SM *et al*. (2019) AKT/mTORC2 inhibition activates FOXO1 function in CLL cells reducing B‐cell receptor‐mediated survival. Clin Cancer Res 25, 1574–1587. 30559170 10.1158/1078-0432.CCR-18-2036 PMC6398589

[108] Seda V , Vojackova E , Ondrisova L , Kostalova L , Sharma S , Loja T , Mladonicka Pavlasova G , Zicha D , Kudlickova Peskova M , Krivanek J *et al*. (2021) FoxO1‐GAB1 axis regulates homing capacity and tonic AKT activity in chronic lymphocytic leukemia. Blood 138, 758–772. 33786575 10.1182/blood.2020008101 PMC8513669

[109] Sharma S , Pavlasova GM , Seda V , Cerna KA , Vojackova E , Filip D , Ondrisova L , Sandova V , Kostalova L , Zeni PF *et al*. (2021) miR‐29 modulates CD40 signaling in chronic lymphocytic leukemia by targeting TRAF4: an axis affected by BCR inhibitors. Blood 137, 2481–2494. 33171493 10.1182/blood.2020005627 PMC7610744

[110] Pavlasova G , Borsky M , Svobodova V , Oppelt J , Cerna K , Novotna J , Seda V , Fojtova M , Fajkus J , Brychtova Y *et al*. (2018) Rituximab primarily targets an intra‐clonal BCR signaling proficient CLL subpopulation characterized by high CD20 levels. Leukemia 32, 2028–2031. 30030508 10.1038/s41375-018-0211-0

[111] Pavlasova G , Borsky M , Seda V , Cerna K , Osickova J , Doubek M , Mayer J , Calogero R , Trbusek M , Pospisilova S *et al*. (2016) Ibrutinib inhibits CD20 upregulation on CLL B cells mediated by the CXCR4/SDF‐1 axis. Blood 128, 1609–1613. 27480113 10.1182/blood-2016-04-709519 PMC5291297

[112] Scheffold A , Jebaraj BMC , Tausch E , Bloehdorn J , Ghia P , Yahiaoui A , Dolnik A , Blätte TJ , Bullinger L , Dheenadayalan RP *et al*. (2019) IGF1R as druggable target mediating PI3K‐δ inhibitor resistance in a murine model of chronic lymphocytic leukemia. Blood 134, 534–547. 31010847 10.1182/blood.2018881029 PMC8212352

[113] Ochodnicka‐Mackovicova K , Bahjat M , Bloedjes TA , Maas C , De Bruin AM , Bende RJ , Van Noesel CJM and Guikema JEJ (2015) NF‐κB and AKT signaling prevent DNA damage in transformed pre‐B cells by suppressing RAG1/2 expression and activity. Blood 126, 1324–1335. 26153519 10.1182/blood-2015-01-621623 PMC4671331

[114] Köhrer S , Havranek O , Seyfried F , Hurtz C , Coffey GP , Kim E , Ten Hacken E , Jäger U , Vanura K , O'Brien S *et al*. (2016) Pre‐BCR signaling in precursor B‐cell acute lymphoblastic leukemia regulates PI3K/AKT, FOXO1 and MYC, and can be targeted by SYK inhibition. Leukemia 30, 1246–1254. 26847027 10.1038/leu.2016.9 PMC5459356

[115] Swaminathan S , Klemm L , Park E , Papaemmanuil E , Ford A , Kweon S‐M , Trageser D , Hasselfeld B , Henke N , Mooster J *et al*. (2015) Mechanisms of clonal evolution in childhood acute lymphoblastic leukemia. Nat Immunol 16, 766–774. 25985233 10.1038/ni.3160 PMC4475638

[116] Shojaee S , Chan LN , Buchner M , Cazzaniga V , Cosgun KN , Geng H , Qiu YH , Von Minden MD , Ernst T , Hochhaus A *et al*. (2016) PTEN opposes negative selection and enables oncogenic transformation of pre‐B cells. Nat Med 22, 379–387. 26974310 10.1038/nm.4062 PMC5178869

[117] Nakae J , Kitamura T , Silver DL and Accili D (2001) The forkhead transcription factor Foxo1 (Fkhr) confers insulin sensitivity onto glucose‐6‐phosphatase expression. J Clin Invest 108, 1359–1367. 11696581 10.1172/JCI12876 PMC209440

[118] Jackson JG , Kreisberg JI , Koterba AP , Yee D and Brattain MG (2000) Phosphorylation and nuclear exclusion of the forkhead transcription factor FKHR after epidermal growth factor treatment in human breast cancer cells. Oncogene 19, 4574–4581. 11030146 10.1038/sj.onc.1203825

[119] Altomonte J , Richter A , Harbaran S , Suriawinata J , Nakae J , Thung SN , Meseck M , Accili D and Dong H (2003) Inhibition of Foxo1 function is associated with improved fasting glycemia in diabetic mice. Am J Physiol Endocrinol Metab 285, E718–E728. 12783775 10.1152/ajpendo.00156.2003

[120] Zhang W , Patil S , Chauhan B , Guo S , Powell DR , Le J , Klotsas A , Matika R , Xiao X , Franks R *et al*. (2006) FoxO1 regulates multiple metabolic pathways in the liver. J Biol Chem 281, 10105–10117. 16492665 10.1074/jbc.M600272200

[121] Haeusler RA , Han S and Accili D (2010) Hepatic FoxO1 ablation exacerbates lipid abnormalities during hyperglycemia. J Biol Chem 285, 26861–26868. 20573950 10.1074/jbc.M110.134023 PMC2930685

[122] Haeusler RA , Hartil K , Vaitheesvaran B , Arrieta‐Cruz I , Knight CM , Cook JR , Kammoun HL , Febbraio MA , Gutierrez‐Juarez R , Kurland IJ *et al*. (2014) Integrated control of hepatic lipogenesis versus glucose production requires FoxO transcription factors. Nat Commun 5, 5190. 25307742 10.1038/ncomms6190 PMC4197140

[123] Langlet F , Haeusler RA , Lindén D , Ericson E , Norris T , Johansson A , Cook JR , Aizawa K , Wang L , Buettner C *et al*. (2017) Selective inhibition of FOXO1 activator/repressor balance modulates hepatic glucose handling. Cell 171, 824–835.e18. 29056338 10.1016/j.cell.2017.09.045 PMC5687849

[124] Lin S , Ptasinska A , Chen X , Shrestha M , Assi SA , Chin PS , Imperato MR , Aronow BJ , Zhang J , Weirauch MT *et al*. (2017) A FOXO1‐induced oncogenic network defines the AML1‐ETO preleukemic program. Blood 130, 1213–1222. 28710059 10.1182/blood-2016-11-750976 PMC5606002

[125] Santamaría CM , Chillón MC , García‐Sanz R , Pérez C , Caballero MD , Ramos F , De Coca AG , Alonso JM , Giraldo P , Bernal T *et al*. (2009) High FOXO3a expression is associated with a poorer prognosis in AML with normal cytogenetics. Leuk Res 33, 1706–1709. 19457552 10.1016/j.leukres.2009.04.024

[126] Sakoe Y , Sakoe K , Kirito K , Ozawa K and Komatsu N (2010) FOXO3A as a key molecule for all‐trans retinoic acid–induced granulocytic differentiation and apoptosis in acute promyelocytic leukemia. Blood 115, 3787–3795. 20215638 10.1182/blood-2009-05-222976

[127] Sykes SM , Lane SW , Bullinger L , Kalaitzidis D , Yusuf R , Saez B , Ferraro F , Mercier F , Singh H , Brumme KM *et al*. (2011) AKT/FOXO signaling enforces reversible differentiation blockade in myeloid Leukemias. Cell 146, 697–708. 21884932 10.1016/j.cell.2011.07.032 PMC3826540

[128] Kurayoshi K , Takase Y , Ueno M , Ohta K , Fuse K , Ikeda S , Watanabe T , Nishida Y , Horike S , Hosomichi K *et al*. (2023) Targeting cis‐regulatory elements of FOXO family is a novel therapeutic strategy for induction of leukemia cell differentiation. Cell Death Dis 14, 642. 37773170 10.1038/s41419-023-06168-2 PMC10541907

[129] Wang F , Demir S , Gehringer F , Osswald CD , Seyfried F , Enzenmüller S , Eckhoff SM , Maier T , Holzmann K , Debatin K‐M *et al*. (2018) Tight regulation of FOXO1 is essential for maintenance of B‐cell precursor acute lymphoblastic leukemia. Blood 131, 2929–2942. 29622548 10.1182/blood-2017-10-813576

[130] Bhansali RS , Rammohan M , Lee P , Laurent AP , Wen Q , Suraneni P , Yip BH , Tsai Y‐C , Jenni S , Bornhauser B *et al*. (2021) DYRK1A regulates B cell acute lymphoblastic leukemia through phosphorylation of FOXO1 and STAT3. J Clin Invest 131, e135937. 33393494 10.1172/JCI135937 PMC7773384

[131] Musilova K and Mraz M (2015) MicroRNAs in B‐cell lymphomas: how a complex biology gets more complex. Leukemia 29, 1004–1017. 25541152 10.1038/leu.2014.351

[132] Hoferkova E , Seda V , Kadakova S , Verner J , Loja T , Matulova K , Skuhrova Francova H , Ondrouskova E , Filip D , Blavet N *et al*. (2024) Stromal cells engineered to express T cell factors induce robust CLL cell proliferation in vitro and in PDX co‐transplantations allowing the identification of RAF inhibitors as anti‐proliferative drugs. Leukemia 38, 1699–1711. 38877102 10.1038/s41375-024-02284-w PMC11286525

[133] Firat E and Niedermann G (2016) FoxO proteins or loss of functional p53 maintain stemness of glioblastoma stem cells and survival after ionizing radiation plus PI3K/mTOR inhibition. Oncotarget 7, 54883–54896. 27448972 10.18632/oncotarget.10702 PMC5342388

[134] Martinez E , Vazquez N , Lopez A , Fanniel V , Sanchez L , Marks R , Hinojosa L , Cuello V , Cuevas M , Rodriguez A *et al*. (2020) The PI3K pathway impacts stem gene expression in a set of glioblastoma cell lines. J Cancer Res Clin Oncol 146, 593–604. 32030510 10.1007/s00432-020-03133-w PMC7391469

[135] Flores D , Lopez A , Udawant S , Gunn B and Keniry M (2023) The foxo1 inhibitor as1842856 triggers apoptosis in glioblastoma multiforme and basal‐like breast cancer cells. FEBS Open Bio 13, 352–362. 10.1002/2211-5463.13547 PMC9900086 36602390

[136] Lee Y‐K , Diaz B , Deroose M , Lee SX , Belvedere S , Accili D , Leibel RL and Lin HV (2021) FOXO1 inhibition synergizes with FGF21 to normalize glucose control in diabetic mice. Mol Metab 49, 101187. 33577983 10.1016/j.molmet.2021.101187 PMC7966865

[137] Bushweller JH (2019) Targeting transcription factors in cancer – from undruggable to reality. Nat Rev Cancer 19, 611–624. 31511663 10.1038/s41568-019-0196-7 PMC8820243

[138] Furuyama T , Kitayama K , Yamashita H and Mori N (2003) Forkhead transcription factor FOXO1 (FKHR)‐dependent induction of PDK4 gene expression in skeletal muscle during energy deprivation. Biochem J 375, 365–371. 12820900 10.1042/BJ20030022 PMC1223677

[139] Matsumoto M , Pocai A , Rossetti L , DePinho RA and Accili D (2007) Impaired regulation of hepatic glucose production in mice lacking the Forkhead transcription factor Foxo1 in liver. Cell Metab 6, 208–216. 17767907 10.1016/j.cmet.2007.08.006

[140] Zhang T , Kim DH , Xiao X , Lee S , Gong Z , Muzumdar R , Calabuig‐Navarro V , Yamauchi J , Harashima H , Wang R *et al*. (2016) FoxO1 plays an important role in regulating β‐cell compensation for insulin resistance in male mice. Endocrinology 157, 1055–1070. 26727107 10.1210/en.2015-1852 PMC4769368

[141] Chen J , Lu Y , Tian M and Huang Q (2019) Molecular mechanisms of FOXO1 in adipocyte differentiation. J Mol Endocrinol 62, R239–R253. 30780132 10.1530/JME-18-0178

[142] Yu W , Chen C and Cheng J (2020) The role and molecular mechanism of FoxO1 in mediating cardiac hypertrophy. ESC Heart Fail 7, 3497–3504. 33089967 10.1002/ehf2.13065 PMC7755013

[143] Xu M , Chen X , Chen D , Yu B and Huang Z (2017) FoxO1: a novel insight into its molecular mechanisms in the regulation of skeletal muscle differentiation and fiber type specification. Oncotarget 8, 10662–10674. 27793012 10.18632/oncotarget.12891 PMC5354690

[144] Teaney NA and Cyr NE (2023) FoxO1 as a tissue‐specific therapeutic target for type 2 diabetes. Front Endocrinol 14, 1286838. 10.3389/fendo.2023.1286838 PMC10629996 37941908

[145] Glauser DA and Schlegel W (2007) The emerging role of FOXO transcription factors in pancreatic β cells. J Endocrinol 193, 195–207. 17470511 10.1677/JOE-06-0191

[146] Talchai C and Accili D (2020) Methods for producing enteroendocrine cells that make and secrete insulin.

[147] Kitamoto T , Lee Y‐K , Sultana N , Watanabe H , McKimpson WM , Du W , Fan J , Diaz B , Lin HV , Leibel RL *et al*. (2022) Chemical induction of gut β‐like‐cells by combined FoxO1/notch inhibition as a glucose‐lowering treatment for diabetes. Mol Metab 66, 101624. 36341906 10.1016/j.molmet.2022.101624 PMC9664469

[148] Belvedere S , Lin HV , DeVita RJ , Turcotte S and Johnstone S (2023) Agents for the treatment of diseases by inhibition of foxo1.

[149] Pavlasova G and Mraz M (2020) The regulation and function of CD20: an “enigma” of B‐cell biology and targeted therapy. Haematologica 105, 1494–1506. 32482755 10.3324/haematol.2019.243543 PMC7271567

[150] Pyrzynska B , Dwojak M , Zerrouqi A , Morlino G , Zapala P , Miazek N , Zagozdzon A , Bojarczuk K , Bobrowicz M , Siernicka M *et al*. (2018) FOXO1 promotes resistance of non‐Hodgkin lymphomas to anti‐CD20‐based therapy. Onco Targets Ther 7, e1423183. 10.1080/2162402X.2017.1423183 PMC5927521 29721381

[151] Morin RD , Assouline S , Alcaide M , Mohajeri A , Johnston RL , Chong L , Grewal J , Yu S , Fornika D , Bushell K *et al*. (2016) Genetic landscapes of relapsed and refractory diffuse large B‐cell lymphomas. Clin Cancer Res 22, 2290–2300. 26647218 10.1158/1078-0432.CCR-15-2123

[152] Blank CU , Haining WN , Held W , Hogan PG , Kallies A , Lugli E , Lynn RC , Philip M , Rao A , Restifo NP *et al*. (2019) Defining ‘T cell exhaustion’. Nat Rev Immunol 19, 665–674. 31570879 10.1038/s41577-019-0221-9 PMC7286441

[153] Weber EW , Maus MV and Mackall CL (2020) The emerging landscape of immune cell therapies. Cell 181, 46–62. 32243795 10.1016/j.cell.2020.03.001 PMC8900215

[154] Chan JD , Scheffler CM , Munoz I , Sek K , Lee JN , Huang Y‐K , Yap KM , Saw NYL , Li J , Chen AXY *et al*. (2024) FOXO1 enhances CAR T cell stemness, metabolic fitness and efficacy. Nature 629, 201–210. 38600376 10.1038/s41586-024-07242-1 PMC11062918

[155] Doan AE , Mueller KP , Chen AY , Rouin GT , Chen Y , Daniel B , Lattin J , Markovska M , Mozarsky B , Arias‐Umana J *et al*. (2024) FOXO1 is a master regulator of memory programming in CAR T cells. Nature 629, 211–218. 38600391 10.1038/s41586-024-07300-8 PMC11062920

[156] Ma MCJ , Tadros S , Bouska A , Heavican T , Yang H , Deng Q , Moore D , Akhter A , Hartert K , Jain N *et al*. (2021) Subtype‐specific and co‐occurring genetic alterations in B‐cell non‐Hodgkin lymphoma. Haematologica 107, 690–701. 10.3324/haematol.2020.274258 PMC8883549 33792219

[157] Reddy A , Zhang J , Davis NS , Moffitt AB , Love CL , Waldrop A , Leppa S , Pasanen A , Meriranta L , Karjalainen‐Lindsberg M‐L *et al*. (2017) Genetic and functional drivers of diffuse large B cell lymphoma. Cell 171, 481–494.e15. 28985567 10.1016/j.cell.2017.09.027 PMC5659841

